# Data Consistency for Data-Driven Smart Energy Assessment

**DOI:** 10.3389/fdata.2021.683682

**Published:** 2021-05-13

**Authors:** Gianfranco Chicco

**Affiliations:** Dipartimento Energia “Galileo Ferraris,” Politecnico di Torino, Torino, Italy

**Keywords:** data-driven, data analytics, machine learning, big data, internet of things, smart energy, knowledge extraction, uncertainty

## Abstract

In the smart grid era, the number of data available for different applications has increased considerably. However, data could not perfectly represent the phenomenon or process under analysis, so their usability requires a preliminary validation carried out by experts of the specific domain. The process of data gathering and transmission over the communication channels has to be verified to ensure that data are provided in a useful format, and that no external effect has impacted on the correct data to be received. Consistency of the data coming from different sources (in terms of timings and data resolution) has to be ensured and managed appropriately. Suitable procedures are needed for transforming data into knowledge in an effective way. This contribution addresses the previous aspects by highlighting a number of potential issues and the solutions in place in different power and energy system, including the generation, grid and user sides. Recent references, as well as selected historical references, are listed to support the illustration of the conceptual aspects.

## Introduction

The present evolution of the electrical systems follows the ideas developed under the *smart grid* paradigm. This paradigm, launched in the first decade of this Millennium in Europe (European Commission, 2006) and in the U.S. (U.S., 2007), deals with the modernisation of the electrical systems by exploiting solutions driven by advanced information and communication technology (ICT) to assist system operation and planning. With the progressive integration of ICT in the smart grid, the power systems are being viewed in the framework of cyber-physical systems (CPS) mostly related to increasingly relevant security aspects (Sridhar et al., 2012), also with the development of corresponding testbeds (Cintuglu et al., 2017).

*Data-driven* approaches are emerging to deal with all the topics referring to the smart grid. The data-driven approach is seen as an alternative to the *model-based* approach, in which computational models are developed by using physical properties and parameters of the modelled system. The model-based approach has been used for many years, with analytical and computational models developed to make simulations and studies on power system operation and planning. However, in recent years many advances occurred in techniques for signal processing and data analytics (Zhang et al., 2018; Bhattarai et al., 2019). These advances are making the data-driven approach more viable and effective, especially because of the independence of the possible approximation of the system model and uncertainty of the parameters used (Musleh et al., 2020). The main advantages and the drawbacks of the model-based and data-driven approaches are summarised in Table 1. It may be seen that there are many opposite characteristics between the two approaches. As such, the choice of whether to adopt a model-based or data-driven approach is often a crucial aspect. A common and practical drawback of the two approaches is the sensibility on the variability in time of data gathered in dynamic conditions.

**Table 1:** Advantages and drawbacks of the model-based and data-driven approaches.

**Approach**	**Advantages**	**Drawbacks**
Model-based	• No historical data required • Limited dependence on data privacy issues • Limited memory needs • No training required • Representative of the physical properties of the system • Useful to simulate extreme or new situations	• System model required • System parameters required • Sensible to model and parameter uncertainty • Possible excessively high computational burden for real-time applications • Need for explicitly modelling non-linearities • Possible divergence • Possible scalability issues • Sensible to the variation in time of dynamic data
Data-driven	• No system model required • No system parameter required • Computational burden (after training) consistent with real-time applications • Useful to discover unknown non-linear characteristics • Scalable	• Historical data required • Data privacy issues • High memory needs • Sensible to measurement accuracy • Training required with appropriate data • Possible overfitting of the training data • Possible lack of representativeness of the physical properties of the system • Limited capabilities for extrapolation and analysis of new situations • Vulnerability of data and exposure to cyber attacks • Sensible to the variation in time of dynamic data

For smart grid applications, Table 2 reports some examples that show limitations of the existing models and possible advances given by the adoption of the data-driven approach.

**Table 2:** Some limitations of existing models and possible data-driven solutions.

**Topic**	**Theoretical approach and limitation**	**Data-driven solutions**
Electricity markets Guo et al. (2020)	Some microeconomic models or game theory are developed under the ideal assumption that the players act in a rational way to maximise their payoffs, using complete information.	In real cases, the players have only incomplete information. Real market data should be used for the analysis of the bidding behaviours.
Demand modelling and forecasting (appliance level) Ji et al. (2020)	Some models try to represent the characteristics of the users and of the appliances by determining suitable probability distributions, for example used within a bottom-up approach Capasso et al., 1994. However, the uncertainty of the behaviour of the individuals and of the external variables, as well as the differences between various types of appliances, are rather difficult to be modelled. Also, the state dynamics of the load are generally not modelled.	Data-driven learning techniques consider the system as a black box and do not require any initial knowledge about the characteristics of the appliances. This avoids the need to describe the real data with probability distributions.
State estimation Weng et al. (2017)	In traditional electrical systems, the estimate of the previous state can be used as an initial value for state estimation, assuming that the system does not change considerably in the short time. However, in a smart grid the generation and consumption may change rapidly, and also frequent changes in the topology lead to fast changes in the states during operation.	The data-driven approach uses historical data to enhance state estimation, provided that sufficient data are available on topologies and measured outcomes recorded for the past operation.
Power system security Tan et al. (2017) and Ruben et al. (2020)	The traditional techniques of analysis used, based on statistical tests, security metrics and state estimation solution with weighted least-squares, may be inadequate to work in case of cleverly conceived false data injection (FDI) attacks.	The adoption of pure data-driven approaches is limited by the scarce availability of real data gathered during security-threatening events.The crucial importance of power system security needs the deployment of hybrid model-based and data-driven solutions for anomaly detection.
Battery storage How et al. (2019)	The operation of battery storage systems is affected by many uncertainties on environmental variables and internal electrochemical variables. All these uncertainties are difficult to be modelled in a highly non-linear and time-dependent model.	A black-box data-driven approach may be useful to represent the complexity of the interactions that occur in the battery system and the corresponding non-linearities.

The next sections are organised to provide a broad view on the nature of the data considered in the smart grid domain. The focus is set on data consistency, highlighting the different forms related to the characteristics and quality of the data and of the information that can be extracted from data. The general aim is to indicate the meaning of the various terms adopted and to provide some insights on specific areas of applications. However, a detailed analysis of the many individual data-driven problems in smart grids and related solution algorithms is outside the scope of this chapter.

The general term referring to the data-driven applications of interest is *learning*, defined as the process with which similarities are identified in a dataset with different inputs. The data-driven approaches used for learning fall into three main categories:

1. *Unsupervised learning*: the algorithm provides the results without any input to verify the validity of the choices. Data are not labelled and are partitioned into groups on the basis of their features by using a suitable computational technique.
2. *Supervised learning*: the algorithm first creates an internal model through specific training in which both input data and the expected outputs are given. Then, the model is used to provide results when new data are given as inputs. The dataset has to be fully representative of the situations analysed, to avoid extrapolations of the results outside of the model created when new input data are provided.
3. *Reinforcement learning*: the operator provides only some directions to affect the learning process. The learning occurs through trial and error, without using training data.

The data-driven approach is useful to discuss the solutions determined from data and measurements referring to real cases (Chen et al., 2017). For this purpose, the data-driven outcomes are directly taken from actual situations and are not affected by theoretical assumptions and hypotheses that could be approximated or over-simplified.

## Data Consistency

Working with a consistent set of data is at the basis of developing appropriate procedures and applications. But what is the meaning of *data consistency*, and how can this concept be quantified? The meaning might depend on the specific domain. Consistency for data communication refers to the possibility of transmitting the data, if needed developing suitable means to allow data exchange among different standards (Kim et al., 2014). Consistency in database systems mostly refers to the fact that data are available, the data format is correct, and the relations with other data are properly preserved. However, this does not mean that the data themselves are correct in terms of representing the phenomenon or process under analysis. For this purpose, data consistency in the specific domain goes beyond the appropriate storage of data in the databases. Manifold aspects characterise data consistency for smart grid applications, as indicated in Table 3 with a conceptual partitioning into data *characteristics*, data *quality*, and *information quality* aspects. These aspects have to be checked before using the data for the specific purposes. With reference to Table 3, the details are addressed in the next subsections.

**Table 3:** Data consistency for smart grid applications.

**Data characteristics**	**Data quality**	**Information quality**
• Presence • Data type • Size • Trust • Certainty • Determinacy	• Resolution • Alignment • Accuracy • Cleanness • Fitting	• Privacy • Completeness • Value

The rationale of the partitioning indicated in Table 3 is as follows:

- *Data characteristics* refer to the origin of the data (“from where”), concerning data structures and representation. Data with a given structure have to be available, non-corrupted, representative of the phenomena studied, and have to reflect significant inputs.
- *Data quality* refers to the data usability (“for which use”), concerning the absence of limitations to the effectiveness of their use in data management procedures.
- *Information quality* concerns the data elaboration (“for what purpose”), to reach meaningful results in the process of transforming data into knowledge.

Data consistency is in many cases taken for granted in model-based approaches, when data could be generated artificially, and in this case would not be affected by real-life issues. However, for data-driven applications it is essential to understand the various causes of non-consistency, trying to mitigate the negative effects of non-consistency whenever possible.

## Data Characteristics

### Presence of the Data

This aspect is related to the absence of missing data. In practise, missing data can be of two types:

1. *Missing values or records*, for which there is no data available. This may bring severe issues, as the absence of data has to be detected and managed in due time, avoiding that the data are stored in a non-regular way.
2. *Flagged missing data*, detected during the data acquisition process, flagging the location in which the data should be saved with an appropriate entry, such as not-a-number (NaN) or conventional values (e.g., negative values when only positive values are meaningful, also using different values to identify different causes of missing data).

After the missing data have been detected and flagged, the need for carrying out further pre-processing actions depends on the usage of the data for the specific applications. In particular:

- No further action is needed if the flagged missing data can be skipped by the procedures of analysis. For example, if the data provided have to be used as regular time series, and the data of multiple users have to be averaged for creating a representative load pattern (Chicco, 2012), it is possible to average at each time step only the available values, calculating the average on different numbers of entries for each time step.
- Data replacement is needed when the integrity of the time series or pattern is essential, e.g., in forecasting procedures, or approaches that transform the original time series into duration curves, in which the relation with time is lost but the presence of missing data would invalidate the meaning of the entire duration curve. Data replacement is carried out with different approaches, depending on the type and location of the missing data. For example, for a time series, when only one or a few data are missing, interpolation algorithms are classically used (e.g., polynomial splines, or based on maximum likelihood with reference to predefined scenarios). Conversely, when many data are missing (for longer periods), the missing data sequence can be reconstructed by adopting prediction tools capable to exploit information on pattern regularities or correlations. Parametric models are used, in which each incomplete attribute is determined by solving a linear regression problem, or the joint probability distribution of all attributes is handled with an expectation maximisation procedure. However, if the relations among attributes is complex, parametric models could be limited in providing effective results (Liu and Zhang, 2021). Thereby, non-parametric models can be used, which do not need to represent relations among the attributes. Most non-parametric methods use artificial neural networks, whereas other solutions have been successfully adopted, such as the principal components pursuit based on the sparse nature of the outliers (Mateos and Giannakis, 2013). Some useful concepts have been based on adopting simultaneous forecasting and backcasting of the missing values (Bokde et al., 2018), exploiting multiple imputation as a way to get many time series, analyse them separately and combining the results (Liu et al., 2018), and considering indicators that represent the re-alignment of the time series in the post-missing data (Chicco et al., 2019). Further solutions combine Extreme Learning Machines with Gaussian Mixture Model (Sovilj et al., 2016), analyse correlation-connected clusters to exploit local correlation among measurements for estimating the missing data (Razavi-Far et al., 2020), apply a denoising convolutional autoencoder (Ryu et al., 2020), or combine statistical and deep learning methods for missing PMU data correction (Zhu and Lin, 2021). The number and variety of the recently proposed methods indicate that the research on missing data imputation for data-driven applications is still very open, in particular to develop applications able to provide corrections close to the real time, which can bring benefits also to other procedures that use the gathered data for different tasks.

### Data Type

Different types of data are involved in the analyses of smart grids. Concerning datasets, data categorisation is essential to set up an effective data pre-processing strategy. Practical experiences in the field have shown that, when data are coming from different sources, data pre-processing could need most of the time dedicated to data analysis. For example, combining the data formats may require time for solving compatibility issues, starting from basic inconsistencies such as the use of decimal dots or commas in the databases, as well as the nature of the field separators and the presence of empty or symbolic entries in a dataset that should contain only numerical values.

The data types can be:

- *Qualitative* (or *categorical*) data, which cannot be measured, and describe the subject by using discrete attributes or values. Qualitative data are further partitioned into nominal (which have no natural ordering) or ordered (for which an internal ranking is possible). Data encoding for qualitative data is a key aspect. Depending on the procedures used, the qualitative information can be handled through numerical data or other labels. Numerical data are typically integer numbers. However, these numbers do not represent the notion of distance among the entries. A particular example of categorical variable is the one that contains the information about date and time, which may have different formats and different extension (from year to sub-second values).
- *Quantitative* data can be represented by integer numbers or real numbers. The quantitative data representation enables the application of a notion of distance, according with a specified metric.

The data exchange in smart grid applications could be critical, because of the variety of data types and formats, and of many interacting individuals. Specific standards have been set up to enable uniformity or at least viable interactions among the products built by different vendors and exploited in different applications (Dong and Kezunovic, 2011).

As a noteworthy example, the Standard IEC 61850 provides ways to exchange information between relays with high-speed communication networks through software, using the same data model for any vendor. The data model is structured in a dedicated way, using objects to describe data for different data sources. Relays are modelled as functions, and in turn functions are modelled as logical nodes. The data encoding follows dedicated rules with specific references for the logical nodes and for the data attributes.

For the example of a substation (Lei et al., 2014), the substation automation system considers different levels (station, bay and process) with different interfacing components:

- Supervisory Control And Data Acquisition (SCADA), which provides an architecture for supervisory management that includes data communication and control devices.
- Intelligent Electronic Devices (IEDs), which communicate with other devices and have some processing capabilities.
- Human Machine Interface (HMI), which includes a dashboard that allows communication between a person and a machine, device or system.
- Merging Unit (MU), which gathers voltage and current signals from the physical system and transform these signals into digital form.

The Standard IEC 61850 uses three specific types of protocols inside the substation:

✓ *Generic Object Oriented Substation Event* (GOOSE), with an *event-driven* real-time communication. When there is a change, the status of the system changes and is immediately updated, and possible commands can be sent. The communication is unconfirmed, to be fast and efficient. No receipt confirmation is asked, and data reliability is guaranteed by repeating the messages at different timings, to avoid losing the packets. The repetition interval is shorter at the beginning and becomes longer during time, until its maximum value is reached.
✓ *Sampled Values*, in which data are sampled and published in a regular way, using an unconfirmed communication, to be faster. The burden to the communication network can be high, and there are limits to the sampling rate and to the number of devices.
✓ *Client/Server* (or Manufacturing Messaging Specification – MMS): uses a confirmed communication, in which the server receives a request from the client and sends a response. It is used to send reports and information that are not time critical. The timing is longer but has to remain acceptable for SCADA. The reports can be sent by the server to the control centre when there is a status change, or when there is a specific request. The objectives are trust and verification of the substation status.

### Size

The present technologies make it possible to extract an enormous amount of information. However, it is important to be able to decide *which* data are gathered, and *how* data are gathered. In fact, if the data are too many with respect to the needs, the analysis could become difficult already at the stages of communicating, storing, viewing, or reading the data.

A recent trend has led to the emergence of the *big data* concept, together with the related “4V's” (Volume, Variety, Velocity, and Veracity), then increased to “5V's” (Volume, Variety, Velocity, Veracity, and Value) (Yin and Kaynak, 2015) that express the main points of data usage.

The term *big data* summarises the possibility of using huge amounts of available data to extract and interpret the knowledge inherent in the processes that generate the data, with different purposes (Hu and Vasilakos, 2016). Big data analytics are used across all the value chain of generation, transmission, distribution, and demand side management (Zhou et al., 2016).

The main aspects of the big data conceptualisation can be related to the smart grid domain, namely:

- *Volume* of information, generally referring to the amount of data handled. For smart grid applications, the rates of the significant events are very different. In some cases, many data with high resolution are needed to sample the electrical variables, for the purpose of representing the details of the phenomena to be analysed (e.g., for power system dynamics or power quality assessment). However, it is also important to avoid the generation of an excessive amount of data when it is not necessary. Having terabytes or more of high-resolution data to handle when the system is operating in quasi steady-state conditions would be highly ineffective. High volumes of data are needed when they come from multiple points and represent aspects to analyse in which comparison of these data or calculation of the correlations is needed (e.g., to study the effects of disturbances in the grid).
- *Velocity* of data generation and processing. In smart grids, the need for handling information gathered in real-time and that need fast elaboration to provide commands requires appropriate data resolution in time.
- *Variety* of data available in structured, semi-structured, and non-structured forms, such that it could be even impossible to store the data into conventional relational databases. It also considers the usage of images or photos, voice, transactional information, or texts. All these aspects correspond to current practises for sending information concerning power system operation and planning.
- *Veracity*, referring to the quality of the data gathered. It depends on working with real data, or with misleading or incorrect information. A particular situation for smart grids is the presence of false data injections that may occur in case of cyber-attacks.
- *Value*, considered as a compromise between costs and benefits for the specific application.

The International research community, in an impetus to endeavour further “V's,” identified other expressions that can be adopted in different contexts to provide a more detailed view on the initial definitions. These further “V's” may have an interpretation in terms of smart grid-related data, namely:

- *Viscosity*, refers to the complexity of the processes that transform data into knowledge. Working with data that have different resolution in time and need non-trivial elaborations to compare them is a form of viscosity. It can also be conditioned by the lack of standardisation of the data formats.
- *Visualisation*, linked to the ways to make the information available to the relevant operators. Data visualisation in the smart grid context is crucial to convey the right information at the right time, especially during alerts in case of contingencies (Sun and Overbye, 2004). The visualisation aspects are quite challenging for large power systems. This leads to a continuing effort to identify new effective ways to reproduce the useful information (Birchfield and Overbye, 2020).
- *Virality*, especially referring to the fast diffusion of data through the Internet, also from social networks. It is a form of velocity, but it is not linked to the mechanism of making the data available. While in general this aspect could be less relevant to smart grids, the developments in progress will further emphasise the delivery of data referring to consumer preferences in local energy systems, such as in the interactions among prosumers within energy communities (Hahnel et al., 2020), or electric vehicles that circulate in the road traffic, for which privacy issues extended from personal data to vehicle location in the Social Internet of Vehicles (Jia et al., 2020).
- *Variability*, particularly relevant by considering the variations in time and unpredictability of many phenomena that occur in the power systems. Variability is a major challenge for data-driven approaches not supported by a physically based model, which only rely upon historical data and tools for data analytics.

The amount of data used in the computational procedures can be reduced by using *data size reduction* (or *data compression*) techniques. The use of compressed data helps reduce the burden on the communication system. In *lossless* compression the initial signal can be reconstructed without losing any point, and the compression depends on how the data are arranged before being transmitted over the communication channels. Conversely, in *lossy* compression a reasonable compromise has to be reached between the data size and the preservation of the information that characterise the data. The latter mainly depends on the type of application, namely, after data compression there are two typical situations:

1. The data can be analysed by using the features available from their compressed forms. This is applied when clustering algorithms are exploited for categorisation of the users' groups (Chicco et al., 2004).
2. The data have to be reconstructed for being used for further applications. In this case, the data compression technique chosen has to enable near-perfect data reconstruction.

The effectiveness of the various techniques for data size reduction also depends on the need and purposes of the possible data reconstruction. For power quality analysis, transform-based coding is applied with a three-stage process, in which the input signal is transformed to obtain uncorrelated coefficients, then scalar or vector quantization is applied to each coefficient, followed by entropy coding (Tcheou et al., 2014). Harmonics-based approaches are useful when some periodicity appears, otherwise wavelet-based approaches or parametric coding with damped sinusoids have been mostly considered. For compression of irregular data, temporal and spatial correlations can be exploited (Stankovic et al., 2013). For electrical load pattern analysis, some techniques, such as singular value decomposition (de Souza et al., 2017), principal component analysis, curvilinear component analysis, and Sammon maps (Chicco et al., 2006), are established to change the nature of the data through mathematical transformations for which an inverse transformation is not defined. The use of shape-related features (Chicco et al., 2003) enables capturing dedicated aspects of the time series during selected groups of hours. Harmonics-based methods are also viable because of some load pattern periodicity (Carpaneto et al., 2006). The wavelet-based approach enables effective data reconstruction through the use of the inverse transform (Ning et al., 2011).

Further solutions have been developed in different directions:

- *Symbolic approximation* is based on the definition of an alphabet of symbols that can be applied after the horizontal and vertical axes have been partitioned depending on the variations of the data along these axes (Notaristefano et al., 2013).
- *Compressive sensing* techniques consider that data are sparse, either in their initial form or after a linear transformation. However, actual data are sparse only to a limited extent, so that the data compression result in approximation errors. The use of dynamic compression schemes could improve the situation, especially if the metric used to quantify sparsity is appropriate, as the coefficient of variation proposed in Joshi et al. (2019).
- *Event-based* approaches aim at identifying the presence of events in the dataset. Event-driven energy metering has been proposed for reducing considerably the amount of data needed to represent load patterns (Simonov et al., 2017c). In the feature-based load data compression proposed in Tong et al. (2016), the generalised extreme value distribution is used to provide the distinction of the load features into base states and load events. Both techniques lead to high data reconstruction effectiveness.
- *Phasor principal component analysis* has been proposed to exploit the correlation between amplitudes and phases of synchrophasor data (Zhang F. et al., 2021).
- *Hybrid* solutions have been considered, such as the combination of wavelet transform, spectral shape estimation with dynamic bit allocation and entropy coding (Cormane and Nascimento, 2016).

### Trust

The notion of *trust* refers to the ability to inspire confidence or faith. The relevant issue is *data corruption*, which could appear in different ways:

- Data coming from *non-trustable sources*: A specific aspect is the possible human intervention to modify the data, in order to hide information, alter the values that could indicate fraudulent behaviour, or building fictitious records to fill existing gaps (e.g., copying a succession of entries of a time series from the same time period of a previous day). When dealing with a massive amount of data, discovering these situations is not easy, however in some cases the data analysis procedures can show strange results that the expert of the domain can interpret by investigating the causes.
- Data corrupted from *deliberate attacks*, such as False Data Injection (FDI) attacks. Cyber-physical attacks are a major issue for smart grid operation, and can be directed to the communication system, the network, or the data (Radoglou-Grammatikis and Sarigiannidis, 2019; Zhang H. et al., 2021). FDI is the most diffuse type of attack acting on the data without affecting the computational codes (Musleh et al., 2020). Denial of service is a type of attack that affects the network by impacting on availability of the service (even when power could be available) because of lack of power supply, control, communication, or data availability (Huseinović et al., 2020; Zhou et al., 2020). Man-in-the-middle is another form of attack that requires specific attention when it is simulated in dedicated platforms (Liu Z. et al., 2020). Time synchronisation attacks affect the specific synchronisation capability of Phasor Measurement Units (PMUs). Preventive measures for mitigating the risks of being damaged by data attacks on PMUs include the introduction of data redundancy from multiple points, or the enhancement of data security. The occurrence of an attack on PMUs can be identified through data-driven methods such as clustering algorithms (Wang X. et al., 2019) or by considering the correlation between the frequency adjustments implemented by the clock and the change in the measured phase angle (Shereen and Dán, 2020). Challenging types of attack act by knowing only data for a limited time period and are able to bypass the bad data detection procedures (Lakshminarayana et al., 2021). Cyber security applications protect devices, networks and data against digital attacks (Sun et al., 2018).

### Certainty: Handling Uncertainties

One of the most challenging aspects for data-driven analysis is the *uncertainty* of the phenomena studied. Different types of uncertainty affect data in the smart grid domain:

- *Environmental*: mostly linked to the generation side, due to the dependence on weather conditions of many types of generation, in particular supplied by renewable energy resources. However, dependency on weather conditions also appears for the demand (e.g., with temperature-dependent loads) and grid components (e.g., with impacts on the thermal rating of overhead lines or cables).
- *Behavioural*: in many cases the users' behaviour and lifestyle has a deep impact on the demand, especially for residential demand, while new behavioural aspects refer to the exploitation of electric vehicles, also with interactions with external uncertainties such as road traffic, and users' preferences.
- *Technical*: referring to the accuracy of the results indicated from measurement systems.
- *Economics-based*: depending on costs and prices, particularly challenging when uncommon behaviours such as price spikes appear. Uncertainty in economic variables also depends on many exogenous variables, most of which refer to external causes that cannot be modelled in a simplified way.

In the treatment of uncertainties, it is possible to make a distinction between *large-scale* and *small-scale* uncertainty (Carpaneto et al., 2011). This distinction is particularly useful for dealing with planning problems and is based on the identification of different time frames:

- When dealing with *large-scale* uncertainty, the time horizon considered is long (e.g., many years), and the random variables that characterise the specific problem (e.g., load patterns, energy prices) could exhibit variations so large that cannot be represented by probability distributions in a meaningful way. In fact, these probability distributions would have very high standard deviations and their mean values could be poorly meaningful. In addition, for some random variables it is requested to represent the trend for future periods, without having references in the past history. For this purpose, *scenario* analyses are most suitable to be considered, in which each scenario is characterised by the evolution of the corresponding random variables, with trends expressed in a more specific way. In a scenario-based approach, different plausible hypotheses and scenarios are tested in order to gain more insights on the potential outcomes of the problem. The scenarios are then weighted according to the preferences of the decision-maker and are studied through approaches based on decision theory (French, 1989) or risk analysis (Miranda and Proença, 1998; Pereira et al., 2000). Besides planning, scenarios are also used in operational problems for those random variables for which it is not simple to set up their values due to high variability in the time steps under analysis. A typical example is the severe uncertainty that can be handled through *information gap decision theory* (IGDT) to exploit the gap between actual and predicted variables, which may come from electricity demand and prices (Soroudi and Ehsan, 2013; Zhao et al., 2017). Another typical case is the wind speed, which could exhibit large variations in hourly intervals (Khazali et al., 2018). Extreme cases deal with the application of IGDT to low-probability high-impact situations found in resilience studies (Salimi et al., 2020).
- When dealing with *small-scale* uncertainty, the operational characteristics of the system (e.g., load patterns, energy prices) are assumed to be known at time steps called elementary time intervals. It is possible to construct groups of random variables whose uncertainty has a relatively low magnitude around the mean value for all the random variables in a given set of elementary time intervals (even not consecutive). In this case, it is possible to construct probabilistic aggregations of random variables for the time intervals belonging to the same group, also taking into account their possible correlations through covariance matrices. On the basis of the probabilistic representation of the random variables, a Monte Carlo approach (with Cholesky factorisation in case of correlated variables) can be used to get the instances of the random variables for each group of elementary time intervals, carrying out probabilistic analysis without executing time-domain simulations.

Another application of the small-scale uncertainty concept is the generation of patterns that represent coupled-in-time evolution of aggregate random variables. An example is the generation of aggregate demand patterns, useful for scenario studies (Sajjad et al., 2015). The random values at different time steps cannot be chosen independently, due to their coupling in time. The construction of a time-coupled probabilistic model of the aggregate residential demand data starts from the available time series of all the individual patterns for a relatively high number of comparable time periods. At each time step, the aggregate demand patterns are constructed, then their cumulative distribution function is determined and is partitioned into a user-defined number of quantiles. Taking the pattern points that fall within the same quantile, the probability distribution of the points reached at the successive time interval is constructed. This procedure is followed for each quantiles and time steps (excluding the last one, for which there is no successive pattern). Small-scale uncertainty is applied inside each quantile. In this way, a reference set of probability distributions is available. It is then possible to pass to the aggregate pattern generation phase, by extracting at random a pattern amplitude at the initial time step, finding the quantile to which it belongs, and select at random the new amplitude from the probability distribution corresponding to that quantile at the following time step. The process is then repeated as a moving window for the successive time steps. It is not strictly needed to use standard probability distributions, as the procedure can be carried out by using the empirical distributions formed with the initial data. With this procedure, many aggregate demand patterns can be generated starting from the same set of initial data. The effectiveness of the results can be assessed by comparing the autocorrelations obtained for demand patterns from initial data-based and simulated datasets.

For addressing uncertainties, the different approaches used are categorised into:

1. *Probabilistic approaches*: the random variables are expressed through probability density functions (PDFs), covariance matrices in case of correlated variables, and cumulative distribution functions (CDFs). Monte Carlo simulations, scenario-based analyses or point estimation methods (Aien et al., 2014) are typically used in these approaches. For these approaches, it is important to identify the PDFs or CDFs of all the relevant variables in an accurate way. Moreover, the effectiveness of the results can be limited when scenario reduction techniques are used for reducing the computational burden. Stochastic programming has been used in various applications, while specific attention is needed to handle data-driven cases in which the historical PDFs are being updated in time (Ding et al., 2018). However, the many random variables and scenarios to be analysed make the computational burden for real-size problems almost intractable (Zio and Aven, 2011). *Chance-constrained* optimisation ensures that the probability that a constraint is satisfied is higher than a given confidence level. If joint chance constraints are considered, the numerical solutions to ensure that the constraints are satisfied overall within a given confidence level could become challenging to solve. Chance-constrained optimisation is a viable solution for problems such as the optimal power flow (OPF), in particular when the uncertainty is bounded inside known constraints and there is a small probability of constraint violation (Baker and Bernstein, 2019) and has been used for solving various problems under different formulations (Roald and Andersson, 2018; Tang et al., 2021). The use of chance constraints becomes challenging when the constraints on the uncertain variables are non-linear.
2. *Possibilistic approaches*: the variables used are represented by using *fuzzy logic* rules (Zadeh, 1965), in which the degree of truth is variable between 0 and 1, and the uncertain variables are represented as fuzzy sets. Many applications to smart grids have been proposed. However, one of the drawbacks of these approaches is the difficulty in assigning appropriate membership functions and degrees of membership.
3. *Interval-based approaches*: in these approaches, *ranges* of input and output variables are used, starting from the basic concepts of *interval analysis*, in which bounds on measurement and rounding errors are considered. The advantage of these approaches for data-driven calculations is that no information about the type of uncertainty of the relevant variables is requested. Interval analysis is suitable to solve linear problems, however for non-linear problems extensions are needed. *Interval arithmetic* is a first step to solve non-linear systems, however it considers independent variations of the uncertain inputs in the corresponding intervals, and as such tends to produce wider and over-conservative ranges of the output variables with respect to the exact ranges. The main issues are the dependency problem (in the presence of intervals considered several times during the calculations, each occurrence is independent and results in an undesired expansion of the intervals in the results) and the wrapping effect (if two variables have a linear relation, in terms of intervals the region to consider becomes a rectangle). *Affine arithmetic* makes the further step of introducing relations between input variables and results. With affine arithmetic, power flow solutions have been proposed to determine the bounds of the power flow solutions by using linear programming Vaccaro et al. (2010), non-linear programming Vaccaro et al. (2013), and the use of polar and rectangular coordinates (Zhang et al., 2017), while recent studies are in progress to deal with interval correlated input random variables (Ran et al., 2020). Further applications of affine arithmetic include the three-phase power flow (Wang et al., 2015), also in integrated transmission and distribution networks (Tang et al., 2020), optimal power flow (Vaccaro and Cañizares, 2017), energy management in microgrids (Romero-Quete and Cañizares, 2019), and the calculation of interval overvoltage risk in distribution systems with distributed energy resources (Wang S. et al., 2020).
4. *Robust optimisation* is a further possibility for handling uncertainties, which does not need to know the probability distributions of the uncertain variables. Robustness may refer to the objective function or to the constraints. In addition, local robustness analysis is carried out in the presence of known boundaries within which the optimal solution has to be found, while non-local robustness analysis should be able to consider also rare events with high impact. Robust optimisation is based on the definition of uncertainty sets. Box-like uncertainty sets can be further elaborated by searching for an internal convex hull (Wang C. et al., 2020). Robust optimisation searches for the solution that performs best in the worst-case scenarios, and could be over-conservative, due to the very low possibility of handling extreme scenarios. Moreover, spatio-temporal correlations can be added to avoid the presence of unreasonable scenarios (Qiu et al., 2021).
5. *Distributionally robust optimization* (DRO): for data-driven applications, the distribution of the uncertain parameters can be observed only through a finite dataset (Zymler et al., 2013; Esfahani and Kuhn, 2018; Cherukuri and Cortés, 2020). The initial assumption is that the exact (unknown) probability distribution belongs to an ambiguity set, which becomes smaller when the number of historical data increases, provided that the data represent comparable situations. The DRO minimises the expected cost in the worst-case over the ambiguity set. An empirical probability distribution can be determined by using the historical data, and the distance between the empirical probability distribution and the exact probability distribution (that belongs to the ambiguity set) can be expressed through the Wasserstein distance (Liu et al., 2020) or the Kullback–Leibler divergence (Chen et al., 2018). DRO has relatively simple requirements on uncertainty with respect to stochastic programming, as well as a simple mathematical tractability. Recent applications refer to optimisation of energy hub operation (Zhao et al., 2020) and determination of bidding strategy models for aggregators (Hajebrahimi et al., 2020).
6. *Model predictive control* (MPC): for data-driven applications, new data may become available during time in dynamic processes. This type of uncertainty cannot be captured by using open-loop optimisation methods; however, it can be handled by MPC methods that perform progressively updated re-optimisation (Huang et al., 2021). On the other side, in MPC the randomness of the uncertain variables depends on forecasts, so that it is not easy to be described. *Stochastic MPC* considers the uncertainty representation to formulate chance constraints (Jiang et al., 2019), trading off between meeting the control objectives and satisfying the probabilistic constraints (Mesbah, 2016). The use of a *distributionally robust MPC* (Huang et al., 2021) can complement the advantages of DRO and MPC, by constructing the ambiguity set by using historical data and recent measurements, then performing re-optimisation by taking into account the forecast errors.
7. *Hybrid approaches* have also been used, in which the variables are of different types. Hybrid stochastic/robust optimisation methods have been used to limit the disadvantages of heavy computational burden of stochastic programming and conservativeness of robust optimisation (Chang et al., 2021). Similarly, hybrid data-driven distributionally robust chance-constrained program has been used for determining a risk-averse offering strategy for a distributed energy resource aggregator (Zhang et al., 2019).

### Determinacy

Determinacy deals with the presence of significant information on the data or the related uncertainties. Lack of significant information is addressed by resorting to different approaches:

- *Intuitionistic fuzzy sets* (IFS): the IFS theory was introduced by Atanassov (1986) as an extension of the fuzzy set theory to add non-determinacy (hesitation) and represent cases in which the fuzzy set theory is not able to use all the information available. Handling together uncertainty and non-determinacy is one of the challenging aspects in the studies on smart grid applications, which can be addressed by following the general principles recalled in Charwand et al. (2020) for electrical load pattern clustering. IFS have been used in power system fault diagnosis to deal with incomplete and uncertain alarm messages (Peng et al., 2018).
- *Rough sets*: rough sets were introduced to deal with uncertainty and vagueness in decision problems (Pawlak, 1982). They have been used in data-driven systems (Pawlak, 1998), including applications with both vagueness and missing data (Kryszkiewicz, 1998), for example for developing fault detection and diagnosis approaches robust to missing data (Ghimire et al., 2018). The rough set theory has also been combined with deep learning for capturing interval knowledge from wind speed time series (Khodayar et al., 2019), and with fuzzy sets to provide solutions to the missing data imputation problem (Amiri and Jensen, 2016).
- *Shadowed and neutrosophic sets*: under the concept of shadowed sets (Pedrycz, 1998), an outcome is represented by using a three-value logic (yes, no, and unknown). Another three-value logic representation is used in the definition of *neutrosophic sets* (Smarandache, 2005).
- *Credal partition* is based on the evidence theory (or belief functions theory), for quantifying the uncertainty for which an input cannot be assigned with certainty to a cluster (Denoeux and Masson, 2004).

The approaches recalled above have not been widely used for smart grid applications yet. However, the related concepts can provide useful insights for future research on data-driven applications.

## Data Quality

### Resolution

Among the different ways to gather data, a significant aspect is the *resolution* (or *granularity*) with which data are available. With reference to data gathered as time series, resolution can be seen in two ways (Chicco, 2010):

- *horizontal* resolution, referring to the way data become available in time:
- *vertical* resolution, referring to the minimum difference with which the amplitude is represented.

Concerning horizontal resolution, data can be represented:

- At regular intervals, such as interval metering. As data are gathered from different sources and in different formats, in a data-driven approach the *alignment* of the data along the coordinates (e.g., time steps) is essential. However, in many cases it is needed to perform some adjustments for aligning the data.
- Event-based, as it happens for different cases:
  *- Triggered events*, used in the power quality analysers, where the relevant point is the ability to identify and characterise the cause of the event on the basis of the data.
  *- Event-driven energy metering* (Simonov, 2013), where the relevant information is the exact representation of the energy between successive events (Simonov et al., 2017b).

About vertical resolution, this issue also depends on the number of digits with which the data are represented within the measurement instrument or the data platform. A typical situation could occur when instruments set up to measure tens of kilowatts are used to measure a few watts in low-loading periods. The modern instrumentation has increased the number of digits, however lower resolution still appears in data loggers scattered in the field.

### Alignment

When data come from different sources, it is possible that the resolutions are not the same. For this purpose, a pre-processing phase is needed to obtain data representations with the same time step. These aspects have been discussed in Chicco et al. (2014a). However, while the data averaging from a shorter time step to a longer one just needs to consider the way the initial data are represented (e.g., stair-wise, or with linear interpolation), if the final time step is shorter than the initial one there is the conceptual limitation that data at shorter time steps generally have greater variability in amplitude during these short time steps. As such, the reconstruction of data with smaller time steps is not conceptually justified, unless specific information on variability at these smaller time steps is known (e.g., from available data for the same user in other periods, or from similar users) and is applied to reconstruct the time series with higher variability.

As an example of data alignment, for a photovoltaic system the useful period of time is from sunrise to sunset, and the corresponding period of time changes day by day. In addition, the ideal conditions for exploiting the photovoltaic system depend on the day of the year, with different maximum solar irradiance that can be reached at clear sky. To ensure comparability among the conditions in which data are used, pre-processing of the solar irradiance data (Chicco et al., 2014b) can be used by normalising:

- The *vertical* (amplitude) axis to make the solar irradiance values comparable. The solar irradiance taken from a clear-sky model (e.g., the Moon-Spencer model, Moon and Spencer, 1942) are used to define a reference solar irradiance pattern for each day. The maximum solar irradiance of each day is used as the normalising factor at that day to re-scale the measured values.
- The *horizontal* (time) axis in order to make the time periods with non-null values (from sunrise to sunset) comparable. The periods from sunrise to sunset are mapped onto the [0,1] range. The corresponding solar irradiance patterns are “stretched” to fit the new horizontal axis. However, the solar irradiance patterns have a different number of points. An interpolation procedure is applied to represent all the patterns in the normalised space with the same number of points.

The reconstructed patterns can then be sent to a clustering procedure, to find out an appropriate grouping of the days (e.g., clear sky and cloudy as the two extreme cases, and a number of intermediate solutions).

### Accuracy

Data accuracy can be addressed by considering different aspects, referring to:

- *Time*, related to data synchronisation, delay and latency. Further insights are reported below.
- *Location*, related to the availability of data gathered in the specific point. Location issues appear in particular with reference to weather data available at meteorological stations, because the location of the measuring devices could be relatively far from the point of interest. For a wind system, relations to correct the wind speed data depending on the location (height) of the anemometers are available, and the local installation of the anemometer is also relevant (Spertino et al., 2012). Solar irradiance data and temperature data can be available closer to the location of interest. However, for solar irradiance sensors the inclination angles and relevant to the appropriateness of the data measured.
- *Amplitude*, affected by the quality of the measurement systems. For direct measurements, the accuracy of the measurement instrument matters. For indirect measurements, the output is affected by the internal errors of the measurement transformers and of the measuring instruments. For solar irradiance sensors, the spectral response has to be similar to those of the photovoltaic cell used, to ensure that the spectral components transformed into electricity correspond to the ones represented by the sensor.
- *Topology*, consisting of correct indication on the grid structure currently in use. In particular, the presence of topology changes has to be promptly communicated, to allow appropriate interpretation of the data gathered from the points that refer to a given grid scheme.

Considering smart grid communication, the communication coverage areas of interest can be partitioned into Wide Area Network (WAN), referring to the utility system, the Neighbourhood Area Network (NAN), corresponding to a portion of the distribution system served by the same transformer, and the Home Area Network (HAN) located at the user's premises (Erol-Kantarci and Mouftah, 2015; Avancini et al., 2019). Data accuracy affects all the data-driven applications for smart grid monitoring and control (Sakis Meliopoulos et al., 2011), PMU-based wide-area measurement systems (De La Ree et al., 2010), quasi-dynamic state estimations in distribution systems (Huang et al., 2015), and distributed control strategies of microgrids (Zhou et al., 2020), just to name a few.

In data-driven assessments, simultaneity among the data referring to the same time instant should be ensured. However, perfect simultaneity cannot be guaranteed, because of the intrinsic *delays* that occur in the data gathering processes. The lower the delays with respect to the dynamics of the problem under analysis, the more the related issues can be disregarded. The Standard IEEE 1588 v2 (also known as Precision Time Protocol, PTP) is a *time synchronisation* technology that enables synchronisation accuracy at the nanosecond level. PTP is used to synchronise the real-time clocks in the nodes of a distributed system that communicate through a network. One of the clocks takes the role of Grandmaster Clock and imposes the time base for the system. The other clocks are managed with a master-slave hierarchy. The event messages need accurate timestamp at both sending and receipt, while general messages do not require timestamps. After a synchronisation event, the slave sends a delay request and receives a response, and on the basis of the available indications calculates the mean propagation delay. In addition to synchronisation, during operation of an application that follows the Standard IEC 61850, data conversion delays make data publishing a bit irregular, and further network delay to the receiver has to be considered.

*Latency* is the time delay between the timestamp of an input data and the timestamp of the same data that reaches an application. For a PMU, the Synchrophasor standard IEEE C37.118.1a (IEEE, 2014) defines the PMU reporting latency as the time difference between the first bit of a PMU report message and the timestamp contained in the report. In WAN monitoring applications based on PMU data, the phasor data concentrator (PDC) has the role of mitigating the latency variations depending on the components of a synchrophasor network. The PDC uses data aggregation to aggregate data sent by many PMUs, and data pushing for sending a time-aligned dataset to the applications (Derviškadić et al., 2018). The *PDC reporting latency* is the relevant quantity that expresses the delay with which a PMU data reaches the application and is given by the sum of the PMU latency, the latency of the communication network, and the PDC latency. Characterising the actual latency of PMU measurements is a challenging open issue (Blair et al., 2019).

For applications to energy management systems, in which a centralised system collects information from the smart metres installed in the local nodes, the *round-trip latency* is the time elapsed from the request of measurements and the completion of the reply from all nodes. The round-trip latency can also serve to determine the maximum number of nodes that a given centralised system can host (Heron et al., 2018). Latency is also relevant to the coordination among real-time simulators located in different sites, used to solve problems in which different networks and hardware-in-the-loop solutions are integrated in the same computational environment (Covrig et al., 2016).

### Cleanness

In the absence of missing data, it is important to cheque whether data are clean, namely, are not affected by noise or by the presence of bad data. The procedures for data cleaning depend on the specific application:

- For applications in which the goal is detecting anomalous conditions (e.g., for power quality purposes), the procedures for identifying noise and bad data have to be accurate enough to avoid confusing true data anomality with problems occurring in the data representation.
- For applications aimed at producing data in normal conditions (e.g., to be used for clustering of load patterns), removing noise and bad data is essential to highlight only the characteristics of the data considered.

A review of data cleansing methods is provided in Chen et al. (2010). Smoothing techniques, among which non-parametric regression, B-spline smoothing, and Kernel smoothing, are recalled, and non-parametric regression is applied to time series that contain outliers and noise, to detect locally corrupted and globally corrupted data with different levels of confidence. Robust non-parametric regression is used in Mateos and Giannakis (2012) with application to electrical load curves. More recent solutions are indicated in Tang et al. (2014) with the introduction of the portrait data, and in El Kababji and Srikantha (2020), among which the Generative Adversarial Networks together with a kernel density estimator are run on individual appliances. The various steps of data pre-processing can be combined a comprehensive approach that includes time synchronisation, noise cleansing, missing data imputation and performance assessment (Martinez-Luengo et al., 2019).

In the data-driven context, also the solution to the classical power flow problem with noisy input data has been addressed, leading to the linearisation of the power flow equations (Liu Y. et al., 2020). Anomaly detection is also applied to short-term load forecasting, with procedures based on robust statistical methods (Chakhchoukh et al., 2011; Guo et al., 2012) and dynamic regression model (Luo et al., 2018). For data denoising, the wavelet decomposition has been mostly applied (Khan et al., 2016).

### Fitting

Data fitting refers to the choice and usage of training data in a supervised learning approach. The aim of the training phase is to learn the relations between inputs and outputs that are embedded in the training data. In general, the use of too many training data is not beneficial, because the training outcomes could try to reproduce to many details of the relation between the data, trying to reach all data points as close as possible. This becomes a disadvantage when the data points are affected by noise. This aspect is denoted as *overfitting*, and results in reducing the possibility of learning the true relation between inputs and outputs. In this way, the relation constructed will not perform well when is used on data different with respect to the ones considered during training. Overfitting tends to construct a relation more complex than what is necessary, and can be avoided in two different ways:

- Use of a *regularisation* term to penalise the cost function considered in the learning process. The penalty term depends on the complexity of the model and drives the solution towards simpler models.
- Application of *early stopping*, by dividing the data used for training into the training set and the validation set, using the latter to cheque the quality of the learning process. In practise, an approximation error is defined as the difference between actual values and predicted values, and the training is stopped when the approximation error decreases for the training set and increases for the validation set. In this way, the identification of the possible poor performance due to overfitting is anticipated in the training phase.

## Information Quality

### Privacy

Privacy is both a limiting factor for data availability and a critical issue concerning the usage of the data by subjects that are gathering the user's data, for the purpose of monitoring the system operation, or for administrative reasons. For protecting the privacy of the users, the observation of the energy consumption data for the purpose of electricity pricing may happen only in the aggregate way (Xu et al., 2018). The objective of reaching any object of our daily life through the Internet of Things (IoT), as well as management of transactions, open challenging issues for privacy preservation (MacDermott et al., 2020). The General Data Protection Regulation (GDPR) harmonises data privacy laws across Europe (European Union, 2016). Preservation of privacy in clustering analysis may be addressed by exploiting the differential privacy concept, by adding random noise in such a way that the true electrical behaviour cannot be identified (Guan et al., 2020).

A typical privacy-related data-driven problem is the identification of the equipment or appliances used in a given system, starting from the data that can be gathered at the supply point of the aggregated load. In Hart (1992) this problem has been given the acronym NALM (Non-intrusive Appliance Load Monitoring), while the acronyms NILM or NIALM are mostly used today with the same meaning. The advantage of non-intrusiveness is the possibility of adopting less hardware (located in a single point of the circuit) and more software, with a final cost-effective balance.

Since its conceptualisation, many approaches have been exploited for understanding how to find effective ways to identify the presence of the loads, and especially which new load appears in the system when a change in the features monitored is detected.

The definition of the features is a fundamental aspect for ensuring the effectiveness of NILM. Among the most used features, it is possible to consider:

- *Active power*, gathered with different time steps. Active power from different loads has the advantage that can be added. However, in general the loads have not a constant power nature, and their power could depend on voltage, which changes during time within a normal operation range that could be up to ±10% of the rated voltage. If the information on the voltage magnitude is available (even though it is not at the load terminals), a voltage-dependent model *P* = *P*_0_ (*V*/*V*_0_)^α^ can be used, where α is a further parameter to be deducted theoretically or experimentally to represent the nature of the load (e.g., α = 0 for constant power, α = 1 for constant current, α = 2 for constant admittance, or other values). Plots of active power vs. reactive power *variations* may enable identification of some appliances (Hart, 1992).
- *Reactive power*, gathered with different time steps. Similar reasoning as above leads to a voltage-dependent model *Q* = *Q*_0_ (*V*/*V*_0_)^β^, where β is a further parameter to be deducted theoretically or experimentally to represent the nature of the load (e.g., β = 0 for constant power, β = 1 for constant current, β = 2 for constant admittance, or other values).
- *Voltage* and *current* RMS values or waveforms, gathered from data sampling at different sampling rates. Voltage and current trajectories are used in Lam et al. (2007) for determining shape features and constructing a taxonomy of the electrical appliances. Detection of spikes, for example the one that could appear when a given appliance is switched on, can provide further knowledge to identify the appliances. In addition to the use of time-domain data, the characterisation of the loads could benefit from the use of frequency-domain data, where specific information is obtained by computing the harmonic spectra through the Fast Fourier Transform. Time-frequency analysis and computation of the wavelet coefficients have been exploited for identifying new features. In Li et al. (2021), time-frequency feature fusion is used for converting one-dimensional time series into two-dimensional images that retain information from the time-frequency domain.

The main differences among the NILM approaches the depend on whether or not there is previous knowledge about the features of the loads:

- The features can be studied online individually for different types of load, by switching on and off one load at a time. In this way, some characteristics of the load “signatures” are identified for constructing an internal library of characteristics that replaces the training process of a NILM solver. However, high differences between the time series of the features for different loads (also for the same load) could appear, depending on the possible controlled load operation, as well as on the user's behaviour in managing the loads.
- A library of initial features can be provided *a priori* (e.g., gathered off-line for each individual load), so that an initial individual online study is not needed. Then, an adaptive process refines the features by using the information about the loads during operation.

Once the features to be used have been selected, the NILM process includes successive stages of data acquisition, event detection, feature extraction, appliance recognition and, when needed, energy estimation. These stages generally contain different aspects, in particular:

- *Data acquisition* occurs through high-frequency sampling, low-frequency sampling at regular time intervals, or in an event-based mode. In the first two cases, the various hardware solutions can be partitioned into low-frequency (1 Hz or less) and high-frequency (over 1 kHz), see Zeifman and Roth (2011). The frequency range from 1 Hz to 1 kHz is considered of interest by Carrie Armel et al. (2013), because in this range data can be provided by smart metres, however suitable algorithms to handle these data have to be developed. In the high-frequency case, better distinction can be achieved also for relatively small appliances that are hard to be identified without availability of more refined information. Event-based NILM is described in Faustine et al. (2021).
- Concerning *event detection*, an event occurs when there is the activation of a new load, or the deactivation of a load already in operation. The challenging part of the event detection is to consider a time step sufficiently short to perform the distinction of the start-up of a single load, without superposition of the start-up of multiple loads. However, as time steps are not synchronised with the beginning of the event, even a steep increase of a relevant feature during the load start-up can be partitioned into successive time steps. Because of this, it is not easy to find sudden changes at successive time steps higher than a given threshold and associate them to a specific load.
- *Feature extraction* depends on the sampling rate, which makes different kinds of data available. When the sampling rate is sufficiently high, the transient characteristics can be measured, which can provide detailed information on the individual signatures of different appliances.
- *Appliance recognition* is carried out by using specific tools, customised with respect to the type of data to be handled. From a probabilistic view, in the presence of the switch-on of a new appliance, the NILM algorithms can be applied to express the probability that the new appliance corresponds to one load or another.
- *Energy estimation* is a relevant aspect for NILM, because it makes it possible to assign the energy consumption to the different types of loads identified. The principles of event-driven energy metering (Simonov et al., 2017a), applied to maintain the actual energy consumption to the reconstructed time series of a load pattern, can be extended to estimate the energy of the individual appliances with the guarantee that the total energy is maintained.

Due to the many aspects referring to NILM, to date there is no ultimate methodology that can give a satisfactory overall response to the load disaggregation problem. The recent availability of publicly available datasets is providing useful common benchmarks for testing different approaches. Pereira and Nunes (2018) review a set of information concerning publicly available datasets, performance metrics, frameworks and toolkits. PLAID (Plug-Load Appliance Identification Dataset) is one of the most used public datasets, with high-frequency sampling (30 kHz) of voltages and currents of different appliances, where the monitored individual and aggregate appliances include switch-on and switch-off instants of the appliances (Medico et al., 2020). In addition to datasets of residential appliances, the recent trend is to extend the analysis to industrial datasets, e.g., LILACD with data gathered at 30 kHz (Kahl et al., 2019), for applying load disaggregation procedures in the context of Industry 4.0.

Further challenges linked to the deployment and evolution of NILM include:

- The use of the information elaborated from NILM for diagnostic purposes, to identify mis-operation cases and failures and assist the development of tools for predictive maintenance.
- The integration of external information, for example on the appliance location in the building, for overcoming the lack of assessment of the appliance location from NILM (which uses only single-point information) and make more information available for enhancing the energy management in buildings without extensive monitoring of all the internal loads, in alternative to IoT-based sensing.
- The construction of a probabilistic framework for addressing the analysis of the data gathered from NILM, which can be integrated with further probabilistic information, e.g., on weather or persons' lifestyle (including possible correlations), to develop more refined models for carrying out energy-related analyses.
- Privacy of NILM data has been addressed by analysing the impact of time granularity (Eibl and Engel, 2015). Privacy of online data has to be guaranteed in emerging applications such as NILM in the cloud (Asres et al., 2021).

### Completeness of the Information

When many data are available, a further issue remains: are data good enough to convey the information needed for the study of interest? Some relevant aspects are:

- Are data *complete* with respect to the purpose of the analysis?
- Are data useful to extract *knowledge* from them?

Concerning the first question, in data-driven approaches incomplete information could have different meanings. The typical example of incomplete information is the missing data concerning bidding in the electricity markets. Even though some information on the market outcomes is available, information about the internal costs of the market players is private and is not disclosed. Another type of incompleteness refers to lack of data on network topology. This is relevant to state estimation, as well as to the fact that for an attacker the information on the network is not known, being private information of the network operator (Liu and Li, 2017; Li and Wang, 2019).

Regarding the extraction of knowledge, the characteristics of the data referring to smart grids are somehow different with respect to other physical systems. Because of the external interactions with the ambient and in case with the users (Wang W. et al., 2019), the collection of many data from different datasets matters. In future applications directed towards smart cities, the data sources will increase in number and type, posing further challenges. The big data platforms developed from information science experts need to be adapted to the smart grid purposes (Tu et al., 2017), to handle a large flow of data from many distributed sources, whose relevance also depends on different time scales.

Transforming data into knowledge is the main goal. The process of knowledge discovery requires the identification of the most effective features considering their complex relations (De Caro et al., 2020). On the information side, the discovery of patterns and relations from datasets is the objective of *data mining* (Hu et al., 2014). However, specific reasoning is needed to interpret the results. *Data analytics* provide different methods to address the management of data to reach specific objectives (Tan et al., 2017):

- *Descriptive analytics*, which describes the past and current system status, visualising synthetic indicators.
- *Predictive analytics*, which exploits models and tools for predicting future trends and estimate the potential risks associated to these trends.
- *Prescriptive analytics*, which exploits advanced decision-making techniques to support the decision process of the user, indicate which are the effects of the decisions, and propose the actions needed to face with possible issues.
- *Automated analytics*, which provides tools to implement in automatic way the actions of interest on the basis of the results.

Data analytics is given the challenging task of transforming data into useful information to be further processed according with specific purposes to extract the relevant knowledge. The expert of the domain has to interact with this process, to avoid that non-meaningful data are processed. Some recently developed fields include:

- The establishment of a data-driven framework for addressing cyber-security, in which there is the convergence of cloud computing and big data analytics to deal with data generation, acquisition, storage and processing, followed by security analytics (Tan et al., 2017).
- The data analytics with strong integration of many data of different nature, for example to extract useful knowledge from the multitude of electric vehicles, including technical and behavioural data, as well as external conditions concerning the traffic, the economics of reaching the parking lots for charging, and the non-scattered location of the charging points (Li et al., 2017).
- The data analytics referring to IoT applications in different contexts, for example to implement transactive energy systems, in which the balance between demand and supply (including storage) to be achieved through the grid is governed by suitable economic mechanisms and control strategies (Zhang Y. et al., 2020). *Edge computing*, in which local tools are able to perform calculations and actions (e.g., on IoT individuals) before connecting to the cloud, provides an interesting prospect for reducing privacy and security issues and alleviate congestion in the various networks (El-Sayed et al., 2018).
- The development of *digital twin* applications, based on the recent success in the manufacturing and automotive industry (Teng et al., 2021). The digital twin is a digital replication of a system in which the last available information is reported. In the power system studies, the networks are traditionally modelled and simulated to understand their response to abnormal events. The novelty brought by the digital twin is to deal with the introduction of IoT and cloud computing in the electrical grid seen as a cyber-physical system (Saad et al., 2020).

A different approach has to be considered when it is requested to apply data-driven analyses to satisfy known objectives or needs. In this case, with the application-driven knowledge (Alahakoon and Yu, 2016), the objectives of stakeholders, business operators, privacy, environmental policies and others are known, and the related analyses can be carried out also with supervised learning tools.

### Value

The value of data has already been indicated among the big data attributes. Besides the economic value of data, already very complicated to determine, the key aspect is the value of the information referring to the data, which goes beyond the economic cost of the data gathering process. Obtaining too many data could have high costs, but only if these data are in a form suitable to be interpreted there can be a benefit for the decision maker. Timeliness of the availability of the information is another crucial aspect. Data analytics has to provide useful results in the due time to be effective. While data quality has been summarised as “fitness for use” (Tayi and Ballou, 1998), the value of the information coming from data elaborated through data analytics is linked to numerous aspects difficult to evaluate, such as understandability, cost effectiveness, competitivity, efficiency and innovation, all of which reflect on the future of the specific business (Mocnej et al., 2021).

In the smart grid context, information quality metrics such as *information age* (i.e., the time elapsed from the local measurement to when the control signal is received) and *mismatch probability* (i.e., the probability that the change of a quantity from the local measurement time to when the control signal is received is lower than a given mismatch interval) have been used for a controlled system (le Fevre Kristensen et al., 2018). These metrics refer to the data themselves and add up to other explicit metrics such as the ratio of errors in the data, or the number of missing values (Koziel et al., 2021). A data quality management system for smart grids can be established by combining different categorised data quality concepts (Ge et al., 2019). However, the definition of an information quality framework is more challenging because of the different meanings the information could have in different contexts of application.

## Final Remarks

A conceptual overview on the nature of the data to be used in data-driven applications relevant in the smart grid context has been presented. Data-driven analyses are based on real data coming from the field, which provide actual information, rather than on hypotheses and assumptions introduced to obtain suitable inputs for theoretical or simulation models. The role of the expert of the domain has been identified as crucial for understanding the correctness of the data. In particular, resorting to the specific expertise is appropriate when a huge amount of data becomes available and it is needed to philtre out bad data, as well as for interpreting the results of the calculations.

The available literature contains many details on aspects that have not been highlighted here. Among them, it is worth mentioning data representation and visualisation, data storage and databases, data processing paradigms for datasets, characterisation of data analytics methodologies, solution methods with machine learning algorithms, computational issues, interfaces, data processing tools (such as clustering for categorisation purposes or outlier detection, data mining, forecasting methods), and performance metrics to quantify data exploitation effectiveness.

The electrical system is subject to an energy transition from the smart grid to the more extended framework of *smart city*, in which extensive measurement, monitoring and surveillance systems lead to collect several data, not limited to technical aspects only. In this way, data-driven procedures could evolve towards knowledge-driven approaches, as already established in different contexts, such as for smart home (Chen et al., 2012) and smart grid (Qiu et al., 2020) applications, and manufacturing systems (Iarovyi et al., 2016; Zhang C. et al., 2020). In the knowledge-driven approach, services, data, and physical component descriptions are addressed by exploiting knowledge of the domain, ontological models, and semantic reasoning. The main challenge is to assess whether the complexity of the power system is not excessive to allow the operators implement knowledge-based applications. Appropriate data-driven procedures may offer opportunities to system operators, users and service providers for identifying the most suitable business models and revenue streams.

## Author Contributions

The author confirms being the sole contributor of this work and has approved it for publication.

## Conflict of Interest

The author declares that the research was conducted in the absence of any commercial or financial relationships that could be construed as a potential conflict of interest.

## Abbreviations

CDF: Cumulative Distribution Function
CPS: Cyber-Physical System
DRO: Distributionally robust optimization
FDI: False Data Injection
GDPR: General Data Protection Regulation
GOOSE: Generic Object Oriented Substation Event
HAN: Home Area Network
HMI: Human Machine Interface
ICT: Information and Communication Technology
IED: Intelligent Electronic Device
IFS: Intuitionistic Fuzzy Set
IGDT: Information Gap Decision Theory
IoT: Internet of Things
MMS: Manufacturing Messaging Specification
MPC: Model Predictive Control
MU: Merging Unit
NAN: Neighbourhood Area Network
NILM: Non-Intrusive Load Monitoring
OPF: Optimal Power Flow
PDC: Phasor Data Concentrator
PDF: Probability Density Function
PMU: Phasor Measurement Unit
PTP: Precision Time Protocol
SCADA: Supervisory Control And Data Acquisition
WAN: Wide Area Network.

## References

[B1] Aien M. Fotuhi-Firuzabad M. Rashidinejad M. (2014). Probabilistic optimal power flow in correlated hybrid wind–photovoltaic power systems. IEEE Trans. Smart Grid 5, 130–138. 10.1109/TSG.2013.2293352

[B2] Alahakoon D. Yu X. (2016). Smart Electricity meter data intelligence for future energy systems: a survey. IEEE Trans. Indust. Inform. 12, 425–436. 10.1109/TII.2015.2414355

[B3] Amiri M. Jensen R. (2016). Missing data imputation using fuzzy-rough methods. Neurocomputing 205, 152–164. 10.1016/j.neucom.2016.04.015

[B4] Asres M. W. Ardito L. Patti E. (2021). Computational cost analysis and data-driven predictive modeling of cloud-based online NILM algorithm. IEEE Trans. Cloud Comput. in press. 10.1109/TCC.2021.3051766

[B5] Atanassov K. (1986). Intuitionistic fuzzy sets. Fuzzy Sets Syst. 20, 87–96. 10.1016/S0165-0114(86)80034-3

[B6] Avancini D. B. Rodrigues J. J. P. C. Martins S. G. B. Rabêlo R. A. L. Al-Muhtadi J. Solic P. (2019). Energy meters evolution in smart grids: a review. J. Clean Prod. 217, 702–715. 10.1016/j.jclepro.2019.01.229

[B7] Baker K. Bernstein A. (2019). Joint chance constraints in ac optimal power flow: improving bounds through learning. IEEE Trans. Smart Grid 10, 6376–6385. 10.1109/TSG.2019.2903767

[B8] Bhattarai B. P. Paudyal S. Luo Y. Mohanpurkar M. Cheung K. Tonkoski R.. (2019). Big data analytics in smart grids: state-of-the-art, challenges, opportunities, and future directions. IET Smart Grid 2, 141–154. 10.1049/iet-stg.2018.0261

[B9] Birchfield A. B. Overbye T. J. (2020). Mosaic packing to visualize large-scale electric grid data. IEEE Open Access J. Power Energy 7, 212–221. 10.1109/OAJPE.2020.3000464

[B10] Blair S. M. Syed M. H. Roscoe A. J. Burt G. M. Braun J. (2019). Measurement and analysis of PMU reporting latency for smart grid protection and control applications. IEEE Access 7, 48689–48698. 10.1109/ACCESS.2019.2903929

[B11] Bokde N. Beck M. W. Martínez Álvarez F. Kulat K. (2018). A novel imputation methodology for time series based on pattern sequence forecasting. Pattern Recogn. Lett. 116, 88–96. 10.1016/j.patrec.2018.09.020 30416234 PMC6223181

[B12] Capasso A. Grattieri W. Lamedica R. Prudenzi A. (1994). A bottom-up approach to residential load modeling. IEEE Trans. Power Syst. 9, 957–964. 10.1109/59.317650

[B13] Carpaneto E. Chicco G. Mancarella P. Russo A. (2011). Cogeneration planning under uncertainty. Part I: multiple time frame approach. Appl. Energy 88, 1059–1067. 10.1016/j.apenergy.2010.10.014

[B14] Carpaneto E. Chicco G. Napoli R. Scutariu M. (2006). Electricity customer classification using frequency-domain load pattern data. Int. J. Elec. Power Energy Syst. 28, 13–20. 10.1016/j.ijepes.2005.08.017

[B15] Carrie Armel K. Gupta A. Shrimali G. Albert A. (2013). Is disaggregation the holy grail of energy efficiency? The case of electricity. Energy Policy 52, 213–234. 10.1016/j.enpol.2012.08.062

[B16] Chakhchoukh Y. Panciatici P. Mili L. (2011). Electric load forecasting based on statistical robust methods. IEEE Trans. Power Syst. 26, 982–991. 10.1109/TPWRS.2010.2080325

[B17] Chang X. Xu Y. Gu W. Sun H. Chow M. Yi Z. (2021). Accelerated distributed hybrid stochastic/robust energy management of smart grids. IEEE Trans. Indust. Inform. 10.1109/TII.2020.3022412

[B18] Charwand M. Gitizadeh M. Siano P. Chicco G. Moshavash Z. (2020). Clustering of electrical load patterns and time periods using uncertainty-based multi-level amplitude thresholding. Int. J. Elec. Power Energy Syst. 117:105624. 10.1016/j.ijepes.2019.105624

[B19] Chen J. Li W. Lau A. Cao J. Wang K. (2010). Automated load curve data cleansing in power systems. IEEE Trans. Smart Grid 1, 213–221. 10.1109/TSG.2010.2053052

[B20] Chen L. Nugent C. D. Wang H. (2012). A knowledge-driven approach to activity recognition in smart homes. IEEE Trans. Knowled. Data Eng. 24, 961–974. 10.1109/TKDE.2011.51

[B21] Chen W. Zhou K. Yang S. Wu C. (2017). Data quality of electricity consumption data in a smart grid environment. Renew. Sustain. Energy Rev. 75, 98–105. 10.1016/j.rser.2016.10.054

[B22] Chen Y. Guo Q. Sun H. Li Z. Wu W. Li Z. (2018). A distributionally robust optimization model for unit commitment based on Kullback–Leibler divergence. IEEE Trans. Power Syst. 33, 5147–5160. 10.1109/TPWRS.2018.2797069

[B23] Cherukuri A. Cortés J. (2020). Cooperative data-driven distributionally robust optimization. IEEE Trans. Automat. Control 65, 4400–4407. 10.1109/TAC.2019.2955031

[B24] Chicco G. (2010). Challenges for smart distribution systems: data representation and optimization objectives, in Proc. 12th International Conference on optimization of electrical and electronic equipment (OPTIM 2010) (Brasov), 1236–1244. 10.1109/OPTIM.2010.5510505

[B25] Chicco G. (2012). Overview and performance assessment of the clustering methods for electrical load pattern grouping. Energy 42, 68–80. 10.1016/j.energy.2011.12.031

[B26] Chicco G. Cocina V. Mazza A. Spertino F. (2014a). Data pre-processing and representation for energy calculations in net metering conditions, in Proc. IEEE Energycon 2014 (Dubrovnik), 413–419. 10.1109/ENERGYCON.2014.6850460

[B27] Chicco G. Cocina V. Spertino F. (2014b). Characterization of solar irradiance profiles for photovoltaic system studies through data rescaling in time and amplitude, in Proc. 49th International Universities' Power Engineering Conference (UPEC 2014) (Cluj-Napoca). 10.1109/UPEC.2014.6934619

[B28] Chicco G. Labate D. Notaristefano A. Piglione F. (2019). Unveil the shape: data analytics for extracting knowledge from smart meters. Energia Elettrica Suppl. J. 96, 1–15. 10.36156/ENERGIA06_01

[B29] Chicco G. Napoli R. Piglione F. (2006). Comparison among clustering techniques for electricity customer classification. IEEE Trans. Power Syst. 21, 933–940. 10.1109/TPWRS.2006.873122

[B30] Chicco G. Napoli R. Piglione F. Postolache P. Scutariu M. Toader C. (2004). Load pattern-based classification of electricity customers. IEEE Trans. Power Syst. 19, 1232–1239. 10.1109/TPWRS.2004.826810

[B31] Chicco G. Napoli R. Postolache P. Scutariu M. Toader C. (2003). Customer characterization options for improving the tariff offer. IEEE Trans. Power Syst. 18, 381–387. 10.1109/TPWRS.2002.807085

[B32] Cintuglu M. H. Mohammed O. A. Akkaya K. Uluagac A. S. (2017). A survey on smart grid cyber-physical system testbeds. IEEE Commun. Surv. Tutorials 19, 446–464. 10.1109/COMST.2016.2627399

[B33] Cormane J. Nascimento F. A de O. (2016). Spectral shape estimation in data compression for smart grid monitoring. IEEE Trans. Smart Grid 7, 1214–1221. 10.1109/TSG.2015.2500359

[B34] Covrig C. F. De Santi G. Fulli G. Masera M. Olariaga M. Bompard E.. (2016). A European Platform for Distributed Real Time Modelling and Simulation of Emerging Electricity Systems. JRC Report EUR 27941 EN, Publications Office of the European Union, Luxembourg, Europe.

[B35] De Caro F. Andreotti A. Araneo R. Panella M. Vaccaro A. Villacci D. (2020). A review of the enabling methodologies for knowledge discovery from smart grids data, in Proc. 2020 IEEE International Conference on Environment and Electrical Engineering and 2020 IEEE Industrial and Commercial Power Systems Europe (EEEIC / IandCPS Europe) (Madrid). 10.1109/EEEIC/ICPSEurope49358.2020.9160678

[B36] De La Ree J. Centeno V. Thorp J. Phadke A. (2010). Synchronized phasor measurement applications in power systems. IEEE Trans. Smart Grid 1, 20–27. 10.1109/TSG.2010.2044815

[B37] de Souza J. C. S. Assis T. M. L. Pal B. C. (2017). Data compression in smart distribution systems via singular value decomposition. IEEE Trans. Smart Grid 8, 275–284. 10.1109/TSG.2015.2456979

[B38] Denoeux T. Masson M. H. (2004). EVCLUS: evidential clustering of proximity data. IEEE Trans. Syst, Man Cybernet 34, 95–109. 10.1109/TSMCB.2002.806496 15369055

[B39] Derviškadić A. Romano P. Pignati M. Paolone M. (2018). Architecture and experimental validation of a low-latency phasor data concentrator. IEEE Trans. Smart Grid 9, 2885–2893. 10.1109/TSG.2016.2622725

[B40] Ding T. Yang Q. Yang Y. Li C. Bie Z. Blaabjerg F. (2018). A data-driven stochastic reactive power optimization considering uncertainties in active distribution networks and decomposition method. IEEE Trans. Smart Grid 9, 4994–5004. 10.1109/TSG.2017.2677481

[B41] Dong Y. Kezunovic M. (2011). Communication infrastructure for emerging transmission-level smart grid applications, in Proceedings of 2011 IEEE Power and Energy Society General Meeting (Detroit, MI). 10.1109/PES.2011.6039640

[B42] Eibl G. Engel D. (2015). Influence of data granularity on smart meter privacy. IEEE Trans. Smart Grid 6, 930–939. 10.1109/TSG.2014.2376613

[B43] El Kababji S. Srikantha P. (2020). A data-driven approach for generating synthetic load patterns and usage habits. IEEE Trans. Smart Grid 11, 4984–4995. 10.1109/TSG.2020.3007984

[B44] El-Sayed H. Sankar S. Prasad M. Puthal D. Gupta A. Mohanty M.. (2018). Edge of things: the big picture on the integration of edge, IoT and the cloud in a distributed computing environment. IEEE Access. 6, 1706–1717. 10.1109/ACCESS.2017.2780087

[B45] Erol-Kantarci M. Mouftah H. T. (2015). Energy-efficient information and communication infrastructures in the smart grid: a survey on interactions and open issues. IEEE Commun. Surv. Tutorials 17, 179–197. 10.1109/COMST.2014.2341600

[B46] Esfahani P. M. Kuhn D. (2018). Data-driven distributionally robust optimization using the Wasserstein metric: performance guarantees and tractable reformulations. Math. Program. 171, 115–166. 10.1007/s10107-017-1172-1

[B47] European Commission (2006). European SmartGrids Technology Platform: Vision and Strategy for Europe's Electricity Networks of the Future. Available online at: http://ec.europa.eu/research/energy/pdf/smartgrids_en.pdf (accessed March 20, 2021).

[B48] European Union (2016). Regulation (EU) 2016/679 of the European Parliament and of the Council of 27 April 2016 on the protection of natural persons with regard to the processing of personal data and on the free movement of such data, and repealing Directive 95/46/EC (General Data Protection Regulation). Off. J. Eur. Union L119. Available online at: https://eur-lex.europa.eu/legal-content/EN/TXT/PDF/?uri=CELEX:32016R0679andfrom=EN (accessed 10 February, 2021).

[B49] Faustine A. Pereira L. Klemenjak C. (2021). Adaptive weighted recurrence graphs for appliance recognition in non-intrusive load monitoring. IEEE Trans. Smart Grid 12, 398–406. 10.1109/TSG.2020.3010621

[B50] French S. (1989). Decision Theory, An Introduction to the Mathematics of Rationality. Chichester, UK: Ellis-Horwood (1989).

[B51] Ge M. Chren S. Rossi B. Pitner T. (2019). Data quality management framework for smart grid systems, in Business Information Systems. BIS 2019. Lecture Notes in Business Information Processing, Vol. 354, eds Abramowicz W. Corchuelo R. (Cham: Springer), 299–310. 10.1007/978-3-030-20482-2_24

[B52] Ghimire R. Zhang C. Pattipati K. R. (2018). A rough set-theory-based fault-diagnosis method for an electric power-steering system. IEEE/ASME Trans. Mechatr. 23, 2042–2053. 10.1109/TMECH.2018.2863119

[B53] Guan Z. Lv Z. Sun X. Wu L. Wu J. Du X.. (2020). A differentially private big data nonparametric Bayesian clustering algorithm in smart grid. IEEE Trans. Netw. Sci. Eng. 7, 2631–2641. 10.1109/TNSE.2020.2985096

[B54] Guo H. Chen Q. Gu Y. Shahidehpour M. Xia Q. Kang C. (2020). A data-driven pattern extraction method for analyzing bidding behaviors in power markets. IEEE Trans. Smart Grid 11, 3509–3521. 10.1109/TSG.2019.2962842

[B55] Guo Z. Li W. Lau A. Inga-Rojas T. Wang K. (2012). Detecting X-outliers in load curve data in power systems. IEEE Trans. Power Syst. 27, 875–884. 10.1109/TPWRS.2011.2167022

[B56] Hahnel U. J. J. Herberz M. Pena-Bello A. Parra D. Brosch T. (2020). Becoming prosumer: revealing trading preferences and decision-making strategies in peer-to-peer energy communities. Energy Policy 137:111098. 10.1016/j.enpol.2019.111098

[B57] Hajebrahimi A. Kamwa I. Abdelaziz M. M. A. Moeini A. (2020). Scenario-wise distributionally robust optimization for collaborative intermittent resources and electric vehicle aggregator bidding strategy. IEEE Trans. Power Syst. 35, 3706–3718. 10.1109/TPWRS.2020.2985572

[B58] Hart G. W. (1992). Nonintrusive appliance load monitoring. Proc. IEEE 80, 1870–1891. 10.1109/5.192069

[B59] Heron J. W. Jiang J. Sun H. Gezerlis V. Doukoglou T. (2018). Demand-response round-trip latency of IoT smartgrid network topologies. IEEE Access 6, 22930–22937. 10.1109/ACCESS.2018.2831254

[B60] How D. N. T. Hannan M. A. Hossain Lipu M. S. Ker P. J. (2019). State of charge estimation for lithium-ion batteries using model-based and data-driven methods: a review. IEEE Access 7, 136116–136136. 10.1109/ACCESS.2019.2942213

[B61] Hu H. Wen Y. Chua T. S. Li X. (2014). Toward scalable systems for big data analytics: A technology tutorial. IEEE Access 2, 652–687. 10.1109/ACCESS.2014.2332453

[B62] Hu J. Vasilakos A. (2016). Energy big data analytics and security: challenges and opportunities. IEEE Trans. Smart Grid 7, 2423–2436. 10.1109/TSG.2016.2563461

[B63] Huang S. Lu C. Lo Y. (2015). Evaluation of AMI and SCADA data synergy for distribution feeder modeling. IEEE Trans. Smart Grid 6, 1639–1647. 10.1109/TSG.2015.2408111

[B64] Huang W. Zheng W. Hill D. J. (2021). Distributionally robust optimal power flow in multi-microgrids with decomposition and guaranteed convergence. IEEE Trans. Smart Grid 12, 43–55. 10.1109/TSG.2020.3012025

[B65] Huseinović A. Mrdović S. Bicakci K. Uludag S. (2020). A survey of denial-of-service attacks and solutions in the smart grid. IEEE Access 8, 177447–177470. 10.1109/ACCESS.2020.3026923

[B66] Iarovyi S. Mohammed W. M. Lobov A. Ferrer B. R. Lastra J. L. M. (2016). Cyber–physical systems for open-knowledge-driven manufacturing execution systems. Proc. IEEE 104, 1142–1154. 10.1109/JPROC.2015.2509498

[B67] IEEE (2014). IEEE Standard for Synchrophasor Measurements for Power Systems-Amendment 1: Modification of Selected Performance Requirements. Standard IEEE C37.118.1a-2014.

[B68] Ji Y. Buechler E. Rajagopal R. (2020). Data-driven load modeling and forecasting of residential appliances. IEEE Trans. Smart Grid 11, 2652–2661. 10.1109/TSG.2019.2959770

[B69] Jia X. Xing L. Gao J. Wu H. (2020). A survey of location privacy preservation in social internet of vehicles. IEEE Access 8, 201966–201984. 10.1109/ACCESS.2020.3036044

[B70] Jiang Y. Wan C. Wang J. Song Y. Dong Z. Y. (2019). Stochastic receding horizon control of active distribution networks with distributed renewables. IEEE Trans. Power Syst. 34, 1325–1341. 10.1109/TPWRS.2018.2879451

[B71] Joshi A. Das L. Natarajan B. Srinivasan B. (2019). A framework for efficient information aggregation in smart grid. IEEE Trans. Indust. Inform. 15, 2233–2243. 10.1109/TII.2018.2866302

[B72] Kahl M. Krause V. Hackenberg R. Haq A. Horn A. Jacobsen H. A.. (2019). Measurement system and dataset for in-depth analysis of appliance energy consumption in industrial environment. Tech. Messen 86, 1–13. 10.1515/teme-2018-0038

[B73] Khan J. Bhuiyan S. Murphy G. Williams J. (2016). Data denoising and compression for smart grid communication. IEEE Trans. Signal Inform. Process. Over Netw. 2, 200–214. 10.1109/TSIPN.2016.2539680

[B74] Khazali A. Rezaei N. Ahmadi A. Hredzak B. (2018). Information gap decision theory based preventive/corrective voltage control for smart power systems with high wind penetration. IEEE Trans. Indust. Inform. 14, 4385–4394. 10.1109/TII.2018.2797105

[B75] Khodayar M. Wang J. Manthouri M. (2019). interval deep generative neural network for wind speed forecasting. IEEE Trans. Smart Grid 10, 3974–3989. 10.1109/TSG.2018.2847223

[B76] Kim D. K. Lee B. Kim S. Yang H. Jang H. Hong D.. (2014). QVT-based model transformation to support unification of IEC 61850 and IEC 61970. IEEE Trans. Power Delivery 29, 598–606. 10.1109/TPWRD.2013.2278848

[B77] Koziel S. Hilber P. Westerlund P. Shayesteh E. (2021). Investments in data quality: evaluating impacts of faulty data on asset management in power systems. Appl. Energy 281:116057. 10.1016/j.apenergy.2020.116057

[B78] Kryszkiewicz M. (1998). Rough set approach to incomplete information systems. Inf. Sci. 112, 39–49. 10.1016/S0020-0255(98)10019-1

[B79] Lakshminarayana S. Kammoun A. Debbah M. Poor H. V. (2021). Data-driven false data injection attacks against power grids: a random matrix approach. IEEE Trans. Smart Grid 12, 635–646. 10.1109/TSG.2020.3011391

[B80] Lam H. Y. Fung G. S. K. Lee W. K. (2007). A novel method to construct taxonomy electrical appliances based on load signaturesof. IEEE Trans. Consum. Electron. 53, 653–660. 10.1109/TCE.2007.381742

[B81] le Fevre Kristensen T. Olsen R. L. Rasmussen J. G. Schwefel H. P. (2018). Information access for event-driven smart grid controllers. Sustain. Energy Grids Netw. 13, 78–92. 10.1016/j.segan.2017.12.005

[B82] Lei H. Singh C. Sprintson A. (2014). Reliability modeling and analysis of IEC 61850 based substation protection systems. IEEE Trans. Smart Grid 5, 2194–2202. 10.1109/TSG.2014.2314616

[B83] Li B. Kisacikoglu M. C. Liu C. Singh N. Erol-Kantarci M. (2017). Big data analytics for electric vehicle integration in green smart cities. IEEE Commun. Magazine 55, 19–25. 10.1109/MCOM.2017.1700133

[B84] Li K. Yin B. Du Z. Sun Y. (2021). A Nonintrusive load identification model based on time-frequency features fusion. IEEE Access 9, 1376–1387. 10.1109/ACCESS.2020.3047147

[B85] Li Y. Wang Y. (2019). False data injection attacks with incomplete network topology information in smart grid. IEEE Access 7, 3656–3664. 10.1109/ACCESS.2018.2888582

[B86] Liu J. Chen Y. Duan C. Lin J. Lyu J. (2020). Distributionally robust optimal reactive power dispatch with wasserstein distance in active distribution network. J. Modern Power Syst. Clean Energy 8, 426–436. 10.35833/MPCE.2019.000057

[B87] Liu T. Wei H. Zhang K. (2018). Wind power prediction with missing data using Gaussian process regression and multiple imputation. Appl. Soft Comput. 71, 905–916. 10.1016/j.asoc.2018.07.027

[B88] Liu X. Li Z. (2017). False data attacks against AC state estimation with incomplete network information. IEEE Trans. Smart Grid 8, 2239–2248. 10.1109/TSG.2016.2521178

[B89] Liu X. Zhang Z. (2021). A two-stage deep autoencoder-based missing data imputation method for wind farm SCADA data. IEEE Sens. J. 21, 10933–10945. 10.1109/JSEN.2021.3061109

[B90] Liu Y. Wang Y. Zhang N. Lu D. Kang C. (2020). A data-driven approach to linearize power flow equations considering measurement noise. IEEE Trans. Smart Grid 11, 2576–2587. 10.1109/TSG.2019.2957799

[B91] Liu Z. Wang Q. Tang Y. (2020). Design of a cosimulation platform with hardware-in-the-loop for cyber-attacks on cyber-physical power systems. IEEE Access 8, 95997–96005. 10.1109/ACCESS.2020.2995743

[B92] Luo J. Hong T. Yue M. (2018). Real-time anomaly detection for very short-term load forecasting. J. Modern Power Syst. Clean Energy 6, 235–243. 10.1007/s40565-017-0351-7

[B93] MacDermott Á. Carr J. Shi Q. Baharon M. R. Lee G. M. (2020). Privacy preserving issues in the dynamic internet of things (IoT), in 2020 International Symposium on Networks, Computers and Communications (ISNCC) (Montreal, QC). 10.1109/ISNCC49221.2020.9297298

[B94] Martinez-Luengo M. Shafiee M. Kolios A. (2019). Data management for structural integrity assessment of offshore wind turbine support structures: data cleansing and missing data imputation. Ocean Eng. 173, 867–883. 10.1016/j.oceaneng.2019.01.003

[B95] Mateos G. Giannakis G. B. (2012). Robust nonparametric regression via sparsity control with application to load curve data cleansing. IEEE Trans. Signal Process. 60, 1571–1584. 10.1109/TSP.2011.2181837

[B96] Mateos G. Giannakis G. B. (2013). Load curve data cleansing and imputation via sparsity and low rank. IEEE Trans. Smart Grid 4, 2347–2355. 10.1109/TSG.2013.2259853

[B97] Medico R. De Baets L. Gao K. Giri S. Kara E. Dhaene T.. (2020). Plaid 2018. Available online at: 10.6084/m9.figshare.10084619.v2 (accessed March 15, 2021).

[B98] Mesbah A. (2016). Stochastic model predictive control: an overview and perspectives for future research. IEEE Control Syst. Mag. 36, 30–44. 10.1109/MCS.2016.2602087

[B99] Miranda V. Proença L. M. (1998). Probabilistic choice vs. risk analysis – conflicts and synthesis. IEEE Trans. Power Syst. 13, 1038–1043. 10.1109/59.709095

[B100] Mocnej J. Pekar A. Seah W. K. G. Papcun P. Kajati E. Cupkova D.. (2021). Quality-enabled decentralized IoT architecture with efficient resources utilization. Robot. Comput. Integr. Manufact. 67:102001. 10.1016/j.rcim.2020.102001

[B101] Moon P. Spencer D. E. (1942). Illumination from a non uniform sky. Trans. Illuminat. Eng. Soc. 37, 707–7261.

[B102] Musleh A. S. Chen G. Dong Z. Y. (2020). A survey on the detection algorithms for false data injection attacks in smart grids. IEEE Trans. Smart Grid 11, 2218–2234. 10.1109/TSG.2019.2949998

[B103] Ning J. Wang J. Gao W. Liu C. (2011). A wavelet-based data compression technique for smart grid. IEEE Trans. Smart Grid 2, 212–218. 10.1109/TSG.2010.2091291

[B104] Notaristefano A. Chicco G. Piglione F. (2013). Data size reduction with symbolic aggregate approximation for electrical load pattern grouping. IET Gener. Transm. Distrib. 7, 108–117. 10.1049/iet-gtd.2012.0383

[B105] Pawlak Z. (1982). Rough sets. Int J. Comput. Inf Sci. 11, 341–356. 10.1007/BF01001956

[B106] Pawlak Z. (1998). Rough set theory and its applications to data analysis. Cybern. Syst. 29, 661–688. 10.1080/019697298125470

[B107] Pedrycz W. (1998). Shadowed sets: representing and processing fuzzy sets. IEEE Trans. Syst. Man Cybernet 28, 103–109. 10.1109/3477.658584 18255928

[B108] Peng H. Wang J. Ming J. Shi P. Pérez-Jiménez M. J. Yu W.. (2018). Fault diagnosis of power systems using intuitionistic fuzzy spiking neural P systems. IEEE Trans. Smart Grid 9, 4777–4784. 10.1109/TSG.2017.2670602

[B109] Pereira L. Nunes N. (2018). Performance evaluation in non-intrusive load monitoring: datasets, metrics, and tools—a review. Wiley Interdiscipl. Rev. Data Min. Knowl. Disc. 8:e1265. 10.1002/widm.1265

[B110] Pereira M. V. F. McCoy M. F. Merrill H. M. (2000). Managing risk in the new power business. IEEE Comput. Appl. Power 13, 19–24. 10.1109/67.831424

[B111] Qiu H. Gu W. Xu X. Pan G. Liu P. Wu Z.. (2021). A historical-correlation-driven robust optimization approach for microgrid dispatch. IEEE Trans. Smart Grid 12, 1135–1148. 10.1109/TSG.2020.3032716

[B112] Qiu J. Chai Y. Tian Z. Du X. Guizani M. (2020). Automatic concept extraction based on semantic graphs from big data in smart city. IEEE Trans. Comput. Soc. Syst. 7, 225–233. 10.1109/TCSS.2019.2946181

[B113] Radoglou-Grammatikis P. I. Sarigiannidis P. G. (2019). Securing the smart grid: a comprehensive compilation of intrusion detection and prevention systems. IEEE Access 7, 46595–46620. 10.1109/ACCESS.2019.2909807

[B114] Ran X. Leng S. Liu K. (2020). A Novel affine arithmetic method with missed the triangular domain with uncertainties. IEEE Trans. Smart Grid 11, 1430–1439. 10.1109/TSG.2019.2938080

[B115] Razavi-Far R. Farajzadeh-Zanjani M. Saif M. Chakrabarti S. (2020). Correlation clustering imputation for diagnosing attacks and faults with missing power grid data. IEEE Trans. Smart Grid 11, 1453–1464. 10.1109/TSG.2019.2938251

[B116] Roald L. Andersson G. (2018). Chance-constrained AC optimal power flow: reformulations and efficient algorithms. IEEE Trans. Power Syst. 33, 2906–2918. 10.1109/TPWRS.2017.2745410

[B117] Romero-Quete D. Cañizares C. A. (2019). An affine arithmetic-based energy management system for isolated microgrids. IEEE Trans. Smart Grid 10, 2989–2998. 10.1109/TSG.2018.2816403

[B118] Ruben C. Dhulipala S. Nagaraj K. Zou S. Starke A. Bretas A.. (2020). Hybrid data-driven physics model-based framework for enhanced cyber-physical smart grid security. IET Smart Grid 3, 445–453. 10.1049/iet-stg.2019.0272

[B119] Ryu S. Kim M. Kim H. (2020). Denoising autoencoder-based missing value imputation for smart meters. IEEE Access 8, 40656–40666. 10.1109/ACCESS.2020.2976500

[B120] Saad A. Faddel S. Youssef T. Mohammed O. A. (2020). On the implementation of iot-based digital twin for networked microgrids resiliency against cyber attacks. IEEE Trans. Smart Grid 11, 5138–5150. 10.1109/TSG.2020.3000958

[B121] Sajjad I. A. Chicco G. Napoli R. (2015). Probabilistic generation of time-coupled aggregate residential demand patterns. IET Gener. Transm. Distrib. 9, 789–797. 10.1049/iet-gtd.2014.0750

[B122] Sakis Meliopoulos A. P. Cokkinides G. Huang R. Farantatos E. Choi S. Lee Y.. (2011). Smart grid technologies for autonomous operation and control. IEEE Trans. Smart Grid 2, 1–10. 10.1109/TSG.2010.2091656

[B123] Salimi M. Nasr M. A. Hosseinian S. H. Gharehpetian G. B. Shahidehpour M. (2020). Information gap decision theory-based active distribution system planning for resilience enhancement. IEEE Trans. Smart Grid 11, 4390–4402. 10.1109/TSG.2020.2992642

[B124] Shereen E. Dán G. (2020). Model-based and data-driven detectors for time synchronization attacks against PMUs. IEEE J. Select. Areas Commun. 38, 169–179. 10.1109/JSAC.2019.2952017

[B125] Simonov M. (2013). Event-driven communication in smart grid. IEEE Commun. Lett. 17, 1061–1064. 10.1109/LCOMM.2013.043013.122798

[B126] Simonov M. Chicco G. Zanetto G. (2017a). Real-time event-based energy metering. IEEE Trans. Indust. Inform. 13, 2813–2823. 10.1109/TII.2017.2680401

[B127] Simonov M. Chicco G. Zanetto G. (2017b). Event-driven energy metering: principles and applications. IEEE Trans. Indust. Appl. 53, 3217–3227. 10.1109/TIA.2017.2679680

[B128] Simonov M. Li H. Chicco G. (2017c). Gathering process data in low voltage systems by enhanced event-driven metering. IEEE Syst. J. 11, 1755–1766. 10.1109/JSYST.2015.2390073

[B129] Smarandache F. (2005). Neutrosophic set – a generalization of the intuitionistic fuzzy set. Int. J. Pure Appl. Mathemat. 24, 287–297. Available online at: https://ijpam.eu/contents/2005-24-3/1/1.pdf (accessed 20 March 2021).

[B130] Soroudi A. Ehsan M. (2013). IGDT based robust decision making tool for DNOs in load procurement under severe uncertainty. IEEE Trans. Smart Grid 4, 886–895. 10.1109/TSG.2012.2214071

[B131] Sovilj D. Eirola E. Miche Y. Björk K. M. Nian R. Akusok A.. (2016). Extreme learning machine for missing data using multiple imputations. Neurocomputing 174, 220–231. 10.1016/j.neucom.2015.03.108

[B132] Spertino F. Di Leo P. Ilie I. S. Chicco G. (2012). DFIG equivalent circuit and mismatch assessment between manufacturer and experimental power-wind speed curves. Renew. Energy 48, 333–343. 10.1016/j.renene.2012.01.002

[B133] Sridhar S. Hahn A. Govindarasu M. (2012). Cyber–Physical system security for the electric power grid. Proc. IEEE, 100, 210–224. 10.1109/JPROC.2011.2165269

[B134] Stankovic V. Stankovic L. Shuang W. Cheng S. (2013). Distributed compression for condition monitoring of wind farms. IEEE Trans. Sustain. Energy 4, 174–181. 10.1109/TSTE.2012.2211047

[B135] Sun C. C. Hahn A. Liu C. C. (2018). Cyber security of a power grid: state-of-the-art. Int. J. Elect. Power Energy Systs. 99, 45–56. 10.1016/j.ijepes.2017.12.020

[B136] Sun Y. Overbye T. J. (2004). Visualizations for power system contingency analysis data. IEEE Trans. Power Syst. 19, 1859–1866. 10.1109/TPWRS.2004.836193

[B137] Tan S. De D. Song W. Yang J. Das S. K. (2017). Survey of security advances in smart grid: a data driven approach. IEEE Commun. Surv. Tutorials 19, 397–422. 10.1109/COMST.2016.2616442

[B138] Tang G. Wu K. Lei J. Bi Z. Tang J. (2014). From landscape to portrait: a new approach for outlier detection in load curve data. IEEE Trans. Smart Grid 5, 1764–1773. 10.1109/TSG.2014.2311415

[B139] Tang K. Dong S. Ma X. Lv L. Song Y. (2021). Chance-constrained optimal power flow of integrated transmission and distribution networks with limited information interaction. IEEE Trans. Smart Grid 12, 821–833. 10.1109/TSG.2020.3021829

[B140] Tang L. Dong S. Zhu C. Song Y. (2020). Affine arithmetic-based coordinated interval power flow of integrated transmission and distribution networks. IEEE Trans. Smart Grid 11, 4116–4132. 10.1109/TSG.2020.2991210

[B141] Tayi G. K. Ballou D. P. (1998). Examining data quality. Commun ACM 41, 54–57. 10.1145/269012.269021

[B142] Tcheou M. P. Lovisolo L. Ribeiro M. V. da Silva E. A. B. Rodrigues M. A. M. Romano J. M. T.. (2014). The compression of electric signal waveforms for smart grids: state of the art and future trends. IEEE Trans. Smart Grid 5, 291–302. 10.1109/TSG.2013.2293957

[B143] Teng S. Y. Touš M. Leong W. D. How B. S. Lam H. L. Máša V. (2021). Recent advances on industrial data-driven energy savings: digital twins and infrastructures. Renew. Sustain. Energy Rev. 135:110208. 10.1016/j.rser.2020.110208

[B144] Tong X. Kang C. Xia Q. (2016). Smart metering load data compression based on load feature identification. IEEE Trans. Smart Grid 7, 2414–2422. 10.1109/TSG.2016.2544883

[B145] Tu C. He X. Shuai Z. Jiang F. (2017). Big data issues in smart grid – a review. Renew. Sustain. Energy Rev. 79, 1099–1107. 10.1016/j.rser.2017.05.134

[B146] U.S. (2007). Energy Independence and Security Act of 2007. The Senate and House of Representatives of the United States of America. Public Law 110–140—Dec. 19, 2007. Available online at: https://www.gpo.gov/fdsys/pkg/PLAW-110publ140/pdf/PLAW-110publ140.pdf (accessed March 20, 2021).

[B147] Vaccaro A. Cañizares C. (2017). An affine arithmetic-based framework for uncertain power flow and optimal power flow studies. IEEE Trans. Power Syst. 32, 274–288. 10.1109/TPWRS.2016.2565563

[B148] Vaccaro A. Cañizares C. Bhattacharya K. (2013). A range arithmetic-based optimization model for power flow analysis under interval Uncertainty. IEEE Trans. Power Syst. 28, 1179–1186. 10.1109/TPWRS.2012.2214405

[B149] Vaccaro A. Cañizares C. Villacci D. (2010). An affine arithmetic-based methodology for reliable power flow analysis in the presence of data uncertainty. IEEE Trans. Power Syst. 25, 624–632. 10.1109/TPWRS.2009.2032774

[B150] Wang C. Gong Z. Liang Y. Wei W. Bi T. (2020). Data-driven wind generation admissibility assessment of integrated electric-heat systems: a dynamic convex hull-based approach. IEEE Trans. Smart Grid 11, 4531–4543. 10.1109/TSG.2020.2993023

[B151] Wang S. Dong Y. Wu L. Yan B. (2020). Interval overvoltage risk based PV hosting capacity evaluation considering PV and load uncertainties. IEEE Trans. Smart Grid 11, 2709–2721. 10.1109/TSG.2019.2960335

[B152] Wang S. Han L. Wu L. (2015). Uncertainty tracing of distributed generations via complex affine arithmetic based unbalanced three-phase power flow. IEEE Trans. Power Syst. 30, 3053–3062. 10.1109/TPWRS.2014.2377042

[B153] Wang W. Chen Q. Gan D. Yang J. Kirschen D. S. Kang C. (2019). Deep learning-based socio-demographic information identification from smart meter data. IEEE Trans. Smart Grid 10, 2593–2602. 10.1109/TSG.2018.2805723

[B154] Wang X. Shi D. Wang J. Yu Z. Wang Z. (2019). Online identification and data recovery for PMU data manipulation attack. IEEE Trans. Smart Grid 10, 5889–5898. 10.1109/TSG.2019.2892423

[B155] Weng Y. Negi R. Faloutsos C. Ilić M. D. (2017). Robust data-driven state estimation for smart grid. IEEE Trans. Smart Grid 8, 1956–1967. 10.1109/TSG.2015.2512925

[B156] Xu Z. Deng T. Hu Z. Song Y. Wang J. (2018). Data-driven pricing strategy for demand-side resource aggregators. IEEE Trans. Smart Grid 9, 57–66. 10.1109/TSG.2016.2544939

[B157] Yin S. Kaynak O. (2015). Big data for modern industry: challenges and trends [Point of View]. Proc. IEEE 103, 143–146. 10.1109/JPROC.2015.2388958

[B158] Zadeh L. A. (1965). Fuzzy sets. Inf. Control 8, 338–353. 10.1016/S0019-9958(65)90241-X

[B159] Zeifman M. Roth K. (2011). Nonintrusive appliance load monitoring: review and outlook. IEEE Trans. Consum. Electr. 57, 76–84. 10.1109/TCE.2011.5735484

[B160] Zhang C. Chen H. Ngan H. Yang P. Hua D. (2017). A mixed interval power flow analysis under rectangular and polar coordinate system. IEEE Trans. Power Syst. 32, 1422–1429. 10.1109/TPWRS.2016.2583503

[B161] Zhang C. Zhou G. Li H. Cao Y. (2020). Manufacturing blockchain of things for the configuration of a data- and knowledge-driven digital twin manufacturing cell. IEEE Int. Things J. 7, 11884–11894. 10.1109/JIOT.2020.3005729

[B162] Zhang F. Wang X. Yan Y. He J. Gao W. Chen G. (2021). A synchrophasor data compression technique with iteration-enhanced phasor principal component analysis. IEEE Trans. Smart Grid 12, 2365–2377. 10.1109/TSG.2020.3046666

[B163] Zhang H. Hu Z. Munsing E. Moura S. J. Song Y. (2019). Data-driven chance-constrained regulation capacity offering for distributed energy resources. IEEE Trans. Smart Grid 10, 2713–2725. 10.1109/TSG.2018.2809046

[B164] Zhang H. Liu B. Wu H. (2021). Smart grid cyber-physical attack and defense: a review. IEEE Access 9, 29641–29659. 10.1109/ACCESS.2021.3058628

[B165] Zhang Y. Huang T. Bompard E. F. (2018). Big data analytics in smart grids: a review. Energy Inform. 1, 1–24. 10.1186/s42162-018-0007-5

[B166] Zhang Y. Krishnan V. V. G. Pi J. Kaur K. Srivastava A. Hahn A.. (2020). Cyber physical security analytics for transactive energy systems. IEEE Trans. Smart Grid 11, 931–941. 10.1109/TSG.2019.2928168

[B167] Zhao J. Wan C. Xu Z. Wang J. (2017). Risk-based day-ahead scheduling of electric vehicle aggregator using information gap decision theory. IEEE Trans. Smart Grid 8, 1609–1618. 10.1109/TSG.2015.2494371

[B168] Zhao P. Gu C. Huo D. Shen Y. Hernando-Gil I. (2020). Two-stage distributionally robust optimization for energy hub systems. IEEE Trans. Indust. Inform. 16, 3460–3469. 10.1109/TII.2019.2938444

[B169] Zhou K. Fu C. Yang S. (2016). Big data driven smart energy management: from big data to big insights. Renew. Sustain. Energy Rev. 56, 215–225. 10.1016/j.rser.2015.11.050

[B170] Zhou Q. Shahidehpour M. Paaso A. Bahramirad S. Alabdulwahab A. Abusorrah A. (2020). Distributed control and communication strategies in networked microgrids. IEEE Commun. Surv. Tutorials 22, 2586–2633. 10.1109/COMST.2020.3023963

[B171] Zhu L. Lin J. (2021). Learning spatio-temporal correlations for missing noisy PMU data correction in smart grid. IEEE Int. Things J. 8, 7589–7599. 10.1109/JIOT.2020.3040195

[B172] Zio E. Aven T. (2011). Uncertainties in smart grids behavior and modeling: what are the risks and vulnerabilities? How to analyze them?. Energy Policy 39, 6308–6320. 10.1016/j.enpol.2011.07.030

[B173] Zymler S. Kuhn D. Rustem B. (2013). Distributionally robust joint chance constraints with second-order moment information. Math. Program. 137, 167–198. 10.1007/s10107-011-0494-7

